# Supplementary material for: Capivasertib enhances chimeric antigen receptor T cell activity in preclinical models of B cell lymphoma
Source: Mol Ther Methods Clin Dev. 2025 Jan 24;33(1):101421. doi: 10.1016/j.omtm.2025.101421 (PMC11850743; doi:10.1016/j.omtm.2025.101421)
Supplement: Document S1. Figures S1–S5 [file mmc1.pdf]

**OMTM, Volume *33***

## Supplemental information

# Capivasertib enhances chimeric antigen receptor T cell activity in preclinical models of B cell lymphoma

**Hui-Ju Hsieh, Ryan Urak, Mary C. Clark, Larry W. Kwak, Stephen J. Forman, and Xiuli Wang**

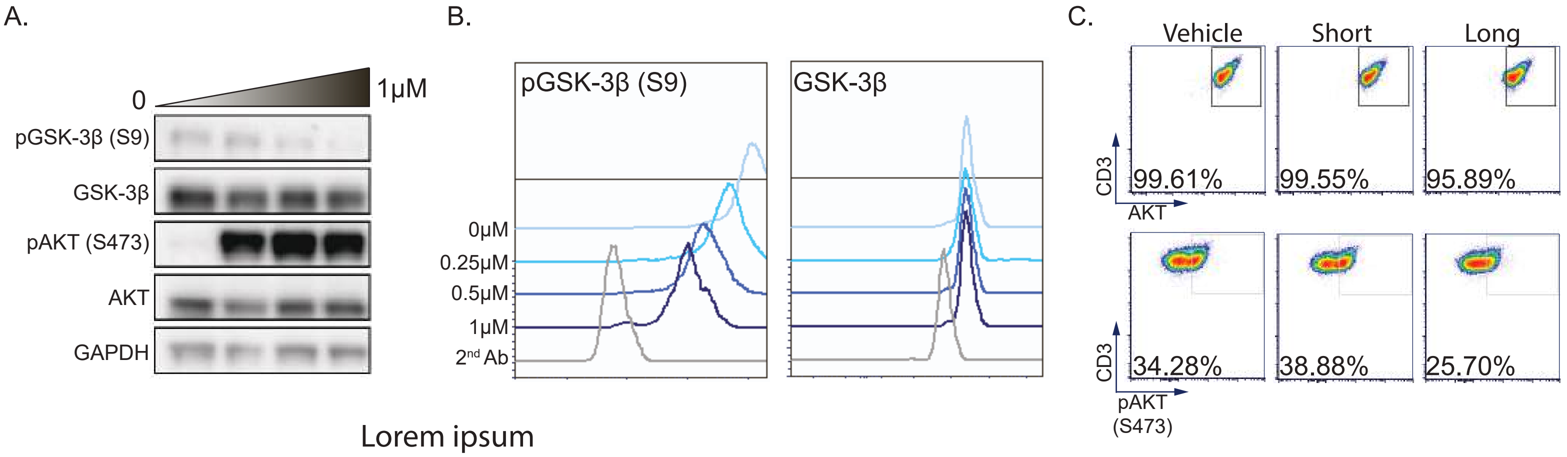


**Figure S1. AKT signaling in CAR T cells was regulated by ex vivo capivasertib treatment.**(A-B) CD19 CAR T cells were expanded for 14 days and treated with various concentrations of capivasertib  for 2 hours: (A) PBMC-derived (0, 0.25, 0.5, 1, and 2 μM) and (B) Tn/mem-derived (0, 0.25, 0.5, and 1 μM). Westernblot and flow cytometry analyses were performed immediately after capivasertib treatment. GAPDH served as the loading control for Western blots, while cells stained only with the secondary antibody were used as flow cytometry controls. (C) The expressions of AKT and pAKT (S473) were evaluated following treatment of CD19 CAR T cells with 0.25 μM capivasertib under short-term and long-term conditions. Control (Ctrl) cells were treated with the same volume of DMSO as the long-term capivasertib-treated group. Data represent n = 4 independent experiments, and error bars denote mean ± SD.

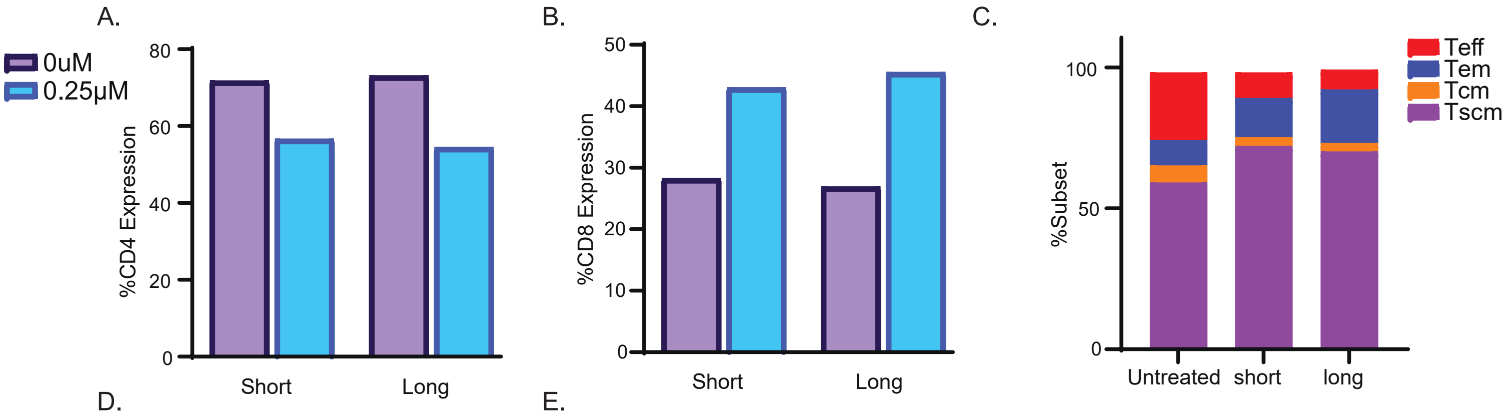


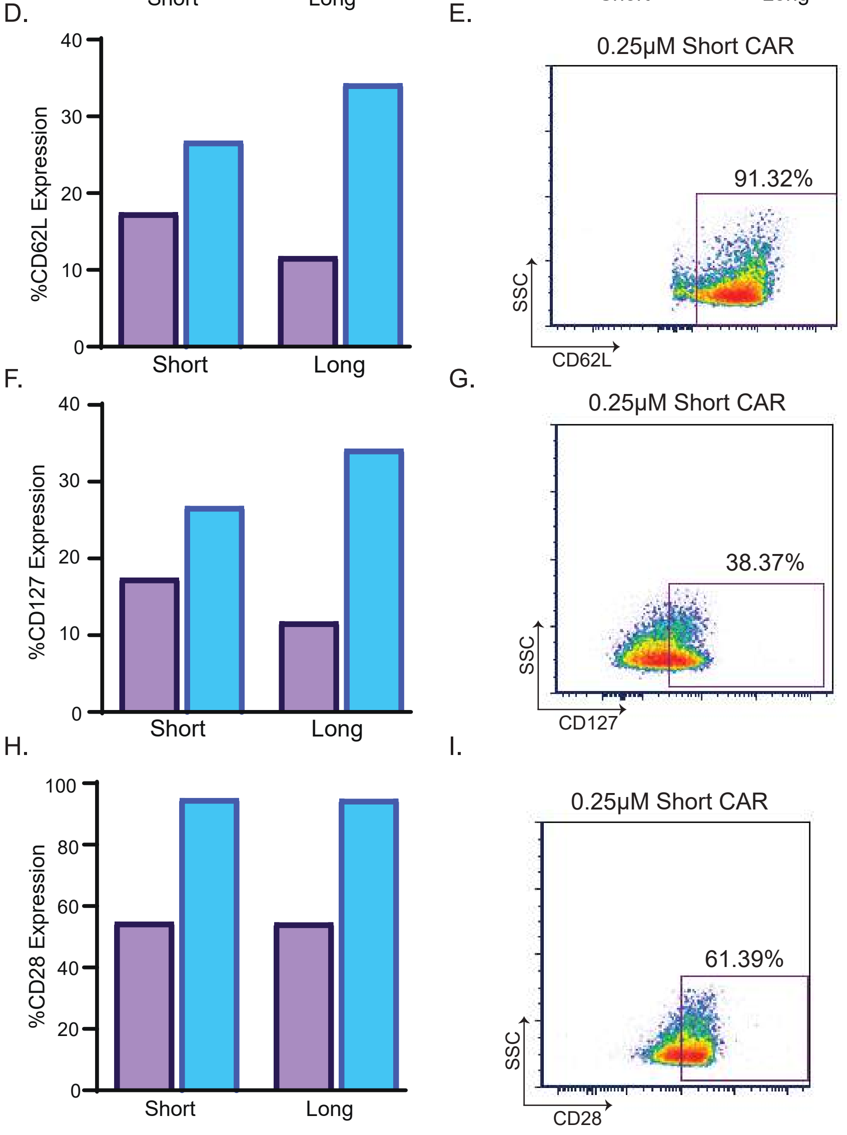


**Figure S2. Ex vivo capivasertib treatment on less differentiated T cells showed phenotypic changes.** The same amounts of cells with Dynabeads were seeded on Day 0 in each group. Dynabeads were removed on Day 7. Cells were expanded by adding fresh media with final concentrations of 50 U/mL of rhIL-2, 0.5 ng/mL of rhIL-15, and capivasertib (0.25μM) and vehicle (0μM) every two days until Day 16. Percent expression of CD4+(A), CD8+(B), T cell subsets (C), and memory markers, CD62L (D-E), CD127 (F-G), CD28 (H-I) on short and long capivasertib treated CAR T cells (CAR gated) at Day 14. n = 4 independent experiments, error bars: mean ± SD, * $p<0.05$, ** $p<0.01$ unpaired t-test. T effector cells (Teff), T effector memory cells (Tem), T central memory cells (Tcm), and T memory stem cells (Tscm).

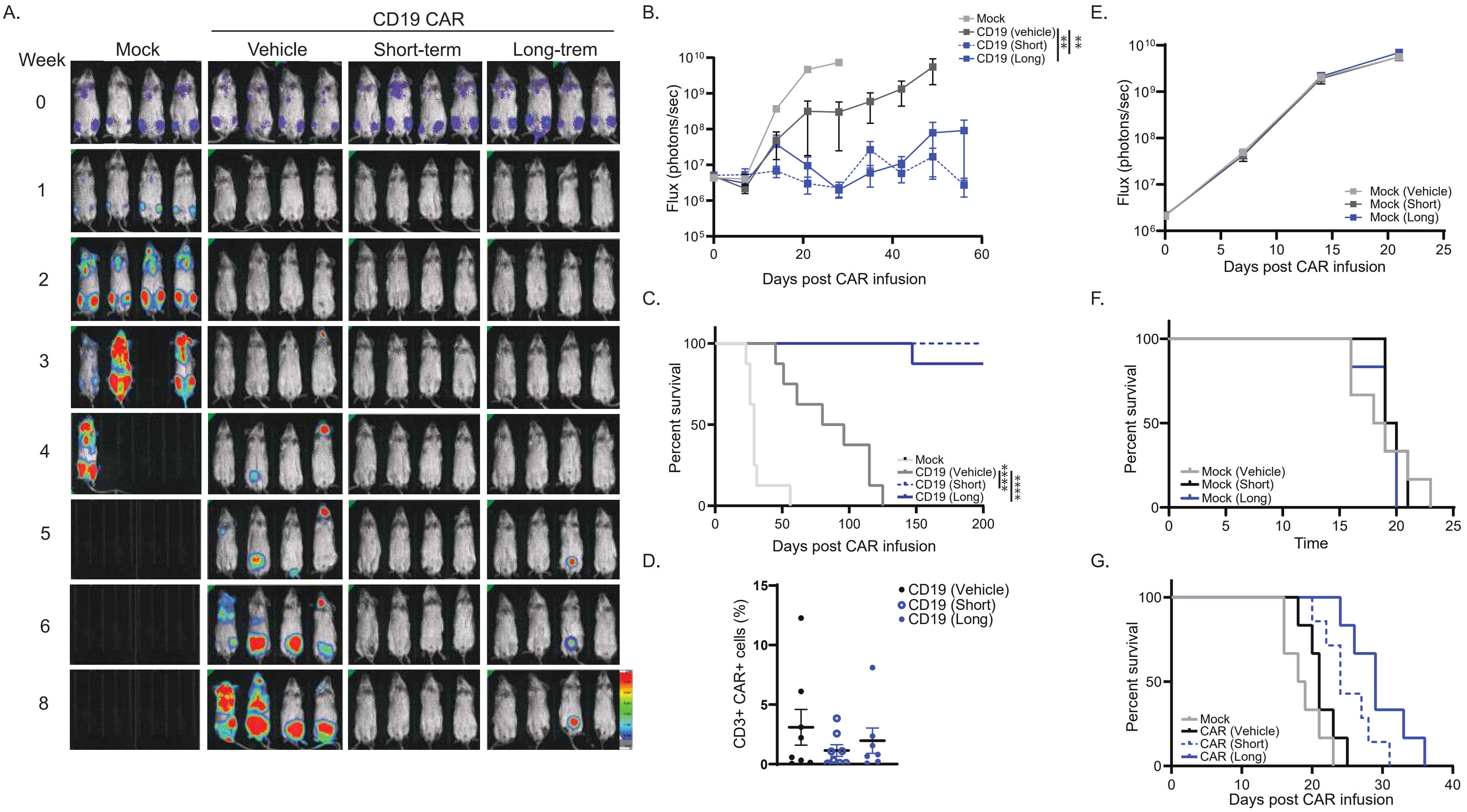


**Figure S3. Ex vivo capivasertib treatment enhances CAR T cells efficacy in vivo** 1 × 10⁶ JeKo-1 cells were injected intravenously into NSG mice at day -7, followed by treatment with 1 × 10⁶ CAR T cells administered intravenously. (A-B) Tumor growth was monitored using live mouse imaging and quantified based on bioluminescent flux. (C) Kaplan-Meier survival curve analysis. (D) Blood samples were collected upon euthanasia to assess CAR T cell persistence. 0.5 × 10⁶ Raji cells were injected intravenously at day -7, and mice were treated with Mock cells consistent with the total amount of T cells in CAR treated experiments intravenously. (E) Tumor growth was analyzed via live mouse imaging and graphed based on flux. (F-G) Kaplan-Meier survival curve analysis was performed. Mock cells were used as a control. Statistical analysis was conducted using the Mann-Whitney test (** $p<0.01$, **** $p < 0.0001$).

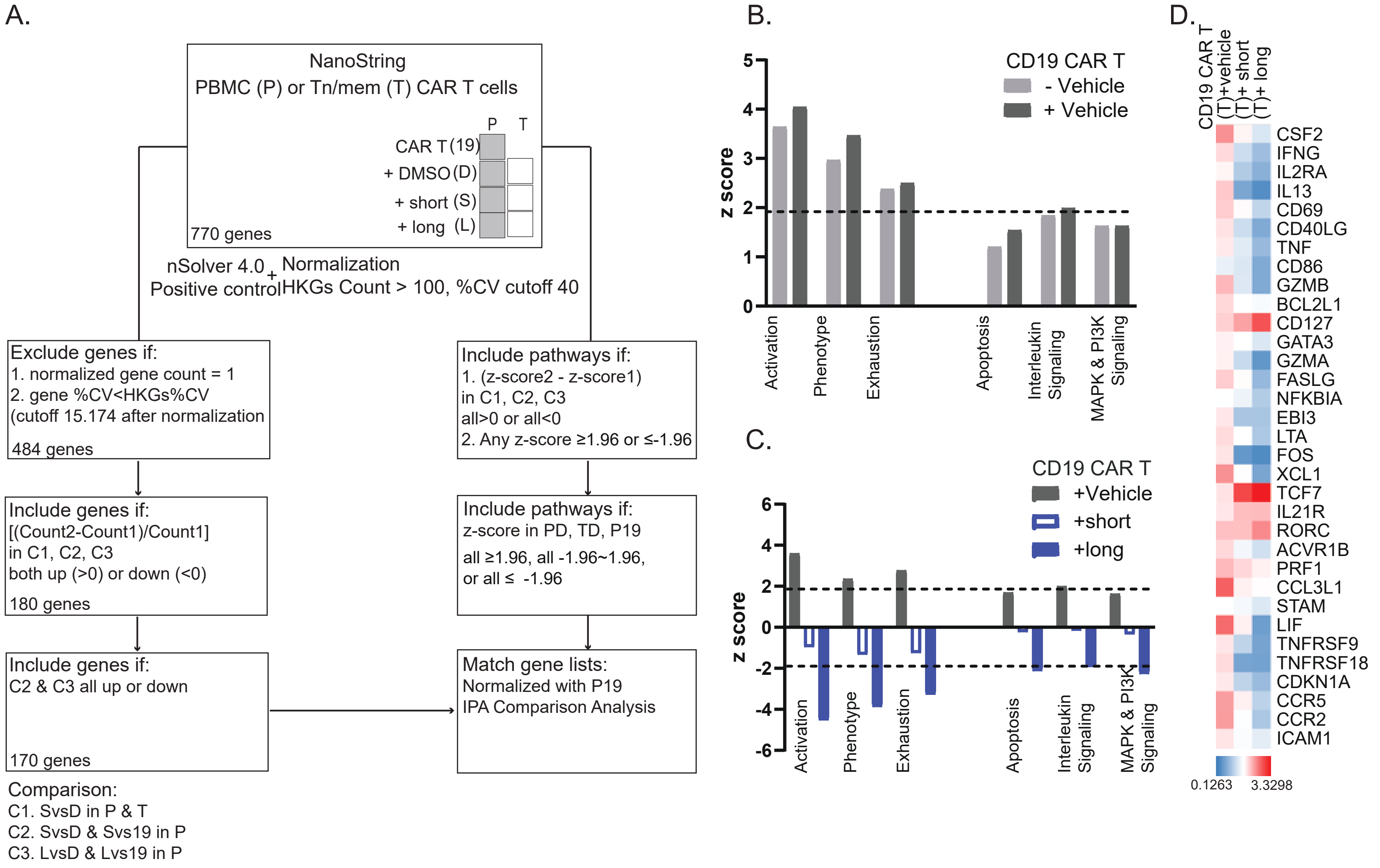


**Figure S4: Ex vivo capivasertib treatment regulated multiple functions of CAR T cells**
(A) The flowchart for NanoString gene expression data analysis. (B) Genes related to specific functions of T cells were analyzed and the dash line indicated z score equaled 1.96. (C) Genes related to specific functions of Tn/mem CAR T cells were analyzed through nCounter Advanced Analysis and the dash lines indicated z-score equaled 1.96 or -1.96. (D) Genes were selected based on the expression changes which were correlated with mouse survival.; n = 4 independent experiments.

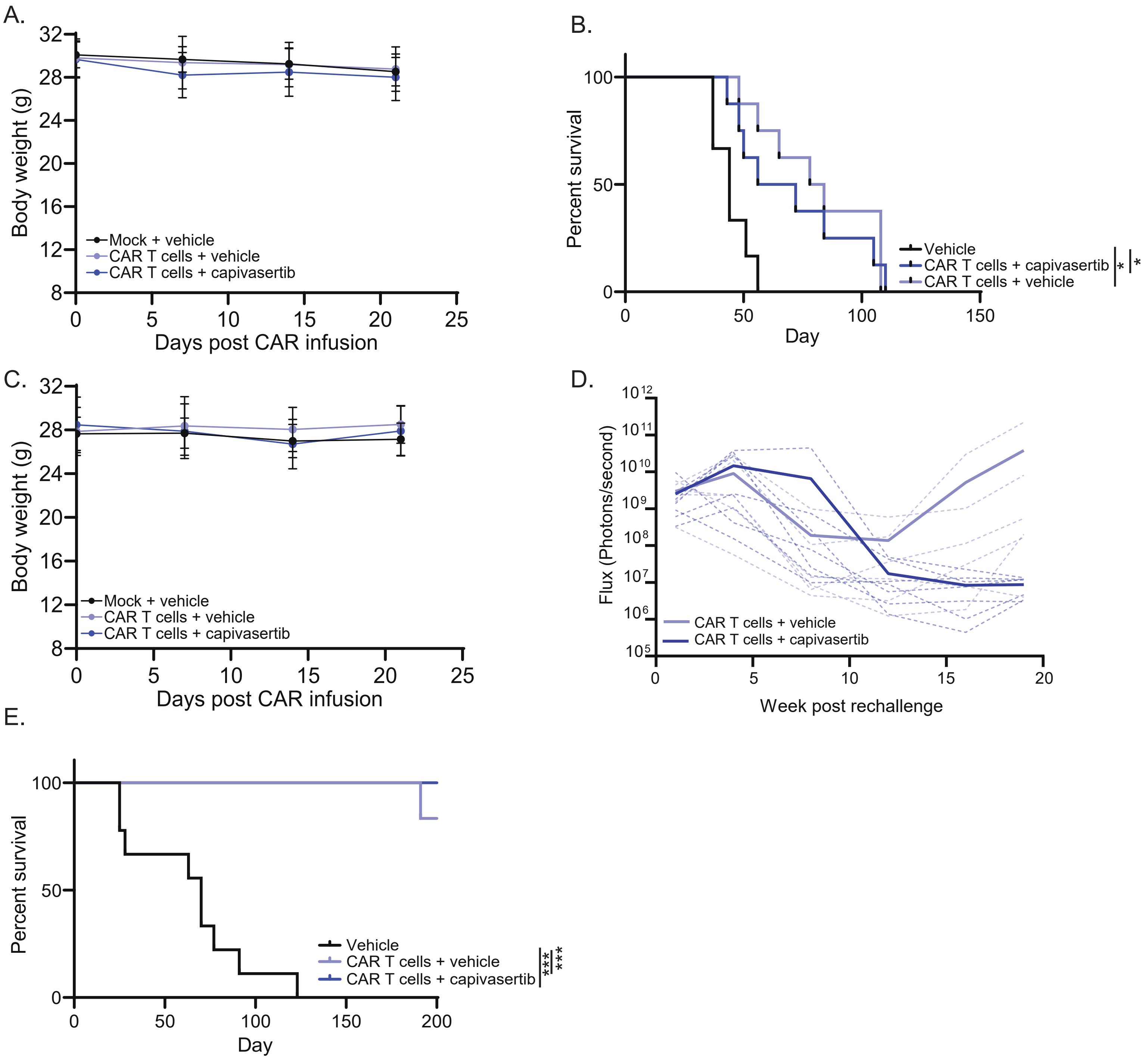


**Figure S5. Combination of capivasertib and CAR T cells prevented relapse in Capiva-sertib susceptible model.** Day 0 marked the initiation of treatment, with mice receiving a single dose of T cells and a 2-week regimen of either vehicle or capivasertib. (A) Mice bearing JeKo-1 cells were weighed once a week for 3 weeks. (B) Kaplan Meier survival curve of capivasertib resistant model (JeKo-1). (C) Mice bearing BJAB cells were weighed once a week for 3 weeks. (D) On day 67, post-tumor engraftment, mice were rechallenged with 2 x $10^6$ capivasertib susceptible BJAB cells intratibially and analyzed for tumor growth by live mice imaging. (E) Kaplan Meier survival cruve. n = 8 (mock T cell groups) & 9 (CAR T cell groups) mice per experiment in the JeKo-1 model and n = 6 & 7 (the CAR T cells + capivasertib group) mice per experiment in the BJAB model, error bar: mean ± SD. * $p<0.05$, *** $p<0.001$.
