# Supplementary material for: Capivasertib enhances chimeric antigen receptor T cell activity in preclinical models of B cell lymphoma
Source: Mol Ther Methods Clin Dev. 2025 Jan 24;33(1):101421. doi: 10.1016/j.omtm.2025.101421 (PMC11850743; doi:10.1016/j.omtm.2025.101421)
Supplement: Document S2. Article plus supplemental information [file mmc2.pdf]

Molecular Therapy
Methods & Clinical Development
Original Article

Capivasertib enhances chimeric antigen receptor T cell activity in preclinical models of B cell lymphoma

Hui-Ju Hsieh,[1,4] Ryan Urak,[1,4] Mary C. Clark,[2] Larry W. Kwak,[3] Stephen J. Forman,[1] and Xiuli Wang[1]

[1]Department of Hematology and Hematopoietic Cell Transplantation, City of Hope, Duarte, CA 91010, USA; [2]Department of Clinical and Translational Project Development, City of Hope, Duarte, CA 91010, USA; [3]Toni Stephenson Lymphoma Center, Department of Hematology and Hematopoietic Cell Transplantation, Beckman Research Institute of City of Hope, Duarte, CA 91010, USA

Phosphatidylinositol 3-kinase (PI3K)/protein kinase B (AKT) signaling is involved in the growth of normal and cancer cells and is crucial for T cell activation. Previously, we have shown that AKT Inhibitor VIII, a selective AKT-1/2 inhibitor, during chimeric antigen receptor (CAR) T cell manufacturing, improves CAR T cell function in preclinical models. Although AKT Inhibitor VIII could enhance CAR T cell function, AKT Inhibitor VIII is not a clinical-grade compound. However, pan-AKT inhibitors have been applied against cancers with *PIK3CA/AKT/PTEN* alterations in clinical trials. We evaluated *ex vivo* and *in vivo* strategies of enhancing CAR T cell therapeutic effect using the pan-AKT inhibitor capivasertib. We found that *ex vivo* 0.25 μM capivasertib treatment during the period of T cell stimulation during manufacture enhanced the antitumor activity of CAR T cells in B cell lymphoma mouse models. Mechanistically, capivasertib changed gene and protein expression patterns related to the functions of memory and effector CAR T cells. Furthermore, *in vivo* combination therapy of capivasertib and CD19-specific CAR T cells led to improved early response to and persistence of functional CAR T cells in mice bearing *PTEN*-deficient lymphoma cells compared to CAR T cells alone. Capivasertib exerts a similar function to AKT Inhibitor VIII in modulating CAR T cells, and combining CAR T cell therapy with capivasertib both *ex vivo* and *in vivo* offers the potential to improve patient outcomes. Since PTEN deficiency is common in cancer and is the main mechanism for capivasertib function, combination therapy may provide an alternative solution for the challenges of CAR T cell therapy.

INTRODUCTION

Chimeric antigen receptor(CAR) therapies have led to unprecedented clinical responses in specific hematological malignancies, including acute lymphoblastic leukemia (ALL) and multiple myeloma (MM).[1–3] Although initial clinical responses are promising and have led to accelerated development of T cell immunotherapies, durability of response and persistence of CAR T cells following treatment remain major limitations across the field. For example, CD19-directed CAR T cells in B cell non-Hodgkin's lymphoma (NHL) induce rates of complete remission (CR) as high as 90% at day 28 post infusion, yet the duration of response at >3 years is only 50%.[4] Similarly, B cell maturation antigen (BCMA)-targeted CAR T cells in MM induce a 70%–80% overall response rate (ORR), with a durability of response of ∼20%–50% at 2 years.[5] These observations suggest that enhancing CAR T cell persistence is key to inducing long-term cures for these diseases. There are several methods under investigation to improve CAR T cell persistence, most of which involve modifying the T cell population used to generate CAR T cells to suppress differentiation and prevent exhaustion.[6–11] To that end, our group has successfully applied the use of cytokines (e.g., interleukin [IL]-15 and -2) during the manufacturing process.[6] However, cytokines alone are likely insufficient to enhance CAR T cell persistence, thus warranting the development of novel methods.

One strategy to extend the persistence of CAR T cells is the use of drugs that modulate cell differentiation and function, including phosphatidylinositol 3-kinase (PI3K)/protein kinase B (AKT) inhibitors.[12–14] PI3K/AKT signaling is a major pathway activated by the engagement of the T cell receptor (TCR), cytokine receptors, and chemokine receptors, and it plays a role in T cell survival, activation, migration, and differentiation.[15–18] We and others have shown that *ex vivo* AKT inhibition during the generation of CAR T cells preserves a less differentiated CAR T cell phenotype, which leads to enhanced persistence and efficacy in preclinical models of B cell malignancies and colorectal cancer.[12,13,19] While emphasizing the promise of AKT inhibition during CAR T cell manufacturing, these studies were performed using a research-grade AKT Inhibitor VIII that cannot be directly translated for clinical use.[13]

Here, we expand upon our previous studies to evaluate the effect of the clinical-grade pan-AKT inhibitor (pAKTi) capivasertib on

Received 30 August 2024; accepted 22 January 2025;
https://doi.org/10.1016/j.omtm.2025.101421.

[4]These authors contributed equally

Correspondence: Xiuli Wang, Department of Hematology and HCT, City of Hope National Medical Center, Duarte, CA 91010, USA.
E-mail: xiuwang@coh.org

© 2025 The Author(s).
Published by Elsevier Inc. on behalf of The American Society of Gene and Cell Therapy.
This is an open access article under the CC BY license (http://creativecommons.org/licenses/by/4.0/).

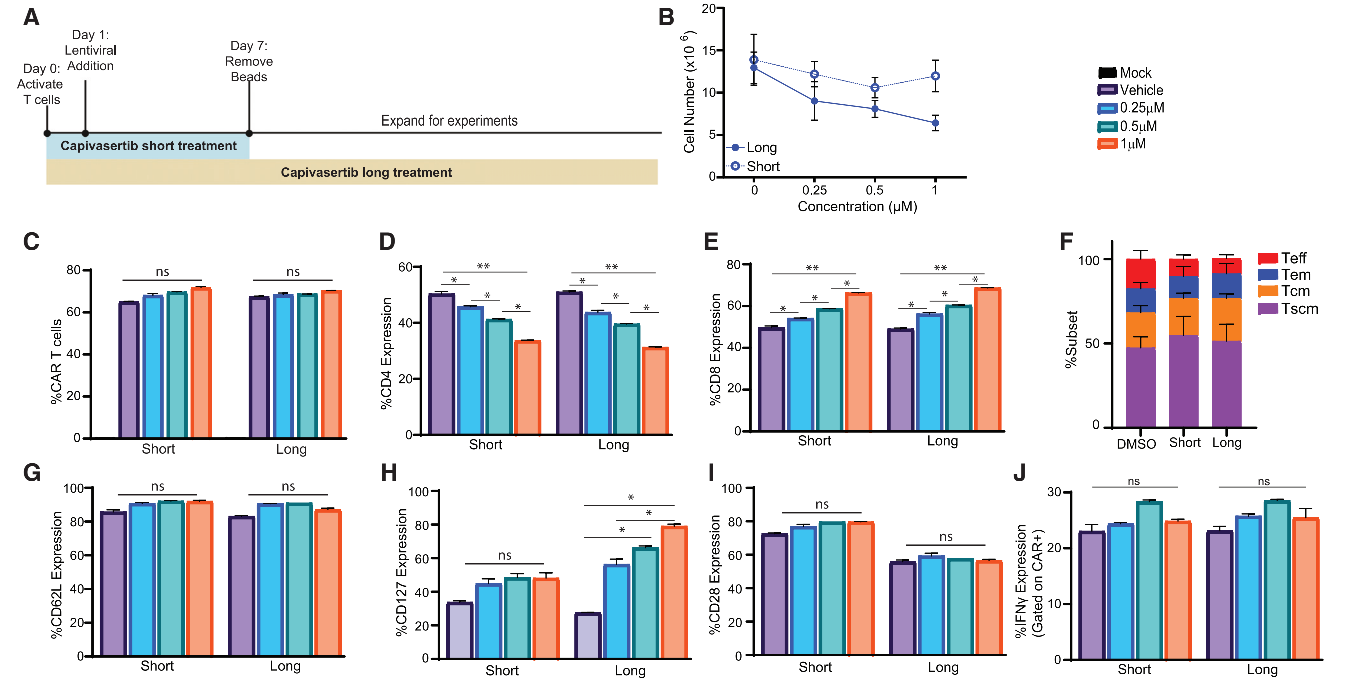

Figure 1. *Ex vivo* capivasertib treatment showed the dose-related phenotypic changes and functional changes in CAR T cells

(A) The outline of *ex vivo* capivasertib (AZD5363) treatment during the CAR T cell manufacturing process. The same amounts of cells with CD3/CD28 Dynabeads were seeded on day 0 in each group. Dynabeads were removed on day 7. Cells were expanded by adding fresh medium with final concentrations of 50 U/mL of rhIL-2, 0.5 ng/mL of rhIL-15, and capivasertib (0.25, 0.5, 1 μM) vehicle (0 μM) every 2 days until day 16. Cells were treated with different concentrations of capivasertib indicated in each panel. (B) Total cell number of viable cells in PBMC-derived CAR T products on day 16. (C) Percentages of CAR T cells were determined based on the truncated EGFR expression on day 7 right after bead removal. Percentage expression of (D) CD4$^+$, (E) CD8$^+$, and (F) T cell subsets, and memory markers (G) CD62L, (H) CD127, and (I) CD28 on short and long capivasertib-treated CAR T cells (CAR gated) at day 14. (J) CAR T cells were cultured with Raji cells with a 1:1 ratio overnight and intracellularly stained for IFN-γ. CAR T cells treated with vehicle (0 μM) are baseline control. n = 4 independent experiments; error bars, mean ± SD; $^*p < 0.05$, $^{**}p < 0.01$, unpaired t test. T effector cells (Teff), T effector memory cells (Tem), T central memory cells (Tcm), and T memory stem cells (Tscm).

CAR T cells, which has not been currently studied. We optimized the use of capivasertib during CAR T cell manufacture and showed enhancement of CAR T cell *in vivo* persistence and antitumor efficacy across multiple CAR T cell platforms and tumor models. Our extensive analysis of gene expression comparing capivasertib-treated vs. control CAR T cells identified enhanced expression of pro-persistence pathways and decreased activation-related pathways following capivasertib treatment. Given the promising clinical development of capivasertib for the treatment of different types of cancer,[20] we interrogated the potential of combining capivasertib and CAR T cells *in vivo*. The combination of capivasertib and CAR T cells resulted in increased CAR T cell persistence and enhanced antitumor efficacy compared to CAR T cell alone. Our study supports both *ex vivo* and *in vivo* applications of capivasertib in enhancing CAR T cell persistence and activity.

RESULTS

Ex vivo capivasertib enhanced memory profiles of CAR T cells without inhibiting function

Given that capivasertib is a pan-AKT inhibitor and could, therefore, have broader effects on CAR T cells compared to the AKT Inhibitor VIII,[13] we tested two different *ex vivo* treatment strategies: short- and long-term treatment (Figure 1A). We administered capivasertib every 48 h during the 7-day bead-activation step only (short-term treatment) or during the entire 16-day manufacturing process (long-term treatment), as we did previously (Figure 1A).[13] For both strategies, we tested a range of capivasertib concentrations (0.25–1 μM) and compared them to vehicle (DMSO) control. Treatment with 2-h capivasertib inhibited phosphorylation of GSK-3β (pGSK-3β), and it induced an increase in phosphorylated AKT (pAKT) (Figures S1A and S1B).[21,22] However, following 14 days of expansion, long-term treatment had slightly dampened pAKT expression (Figure S1C). Following 16 days of expansion, we observed short-term treatment did not impact T cell growth, while long-term capivasertib resulted in a dose-dependent decrease in T cell growth (Figure 1B). CAR expression was unaffected by short- or long-term capivasertib treatment (Figure 1C), but there was a statistically different dose-dependent decrease in CD4+ cells and an increase in CD8+ cells under both treatment strategies (Figures 1D and 1E). Since capivasertib inhibition may impede T cell differentiation,[13] we evaluated the distribution of T cell subsets, by analysis of CD45RA and CD62L expression, at the end of manufacturing and observed that both short- and long-term capivasertib modestly

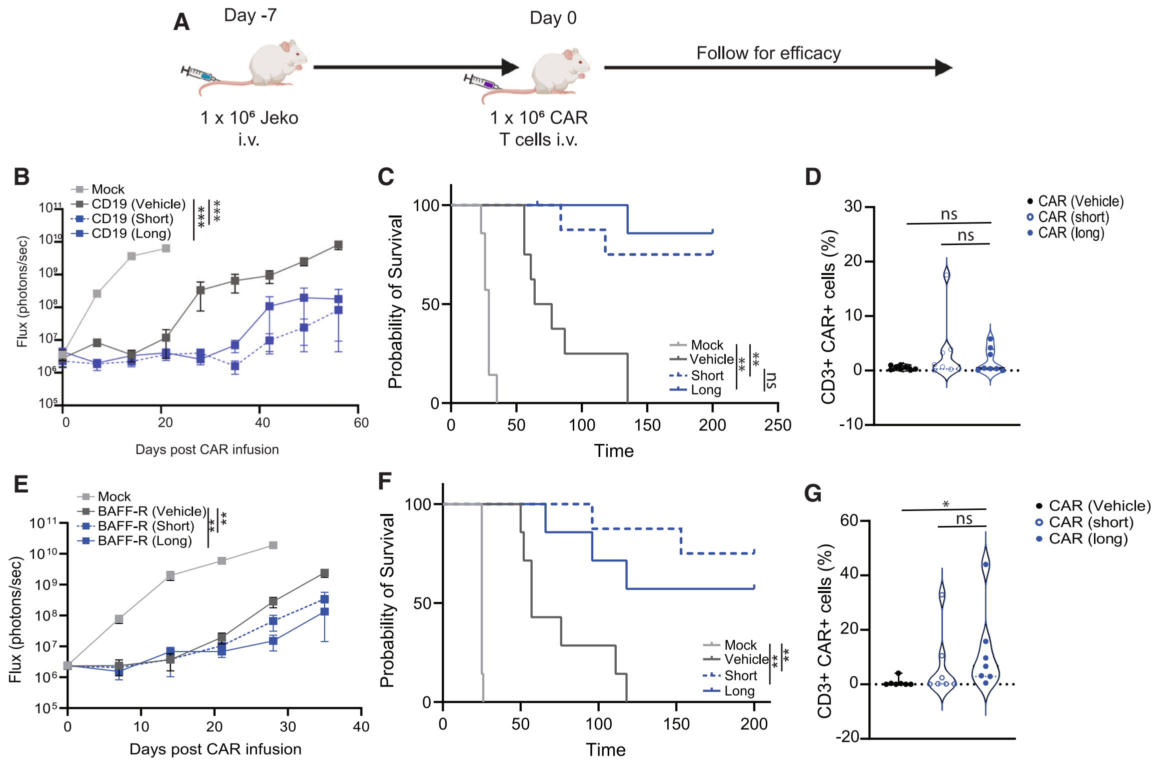

Figure 2. *Ex vivo* capivasertib improved persistence of Tn/mem-derived CAR T cells *in vivo*

(A) The schema of the JeKo-1 xenograft mouse model, in which we injected 1×10^6 JeKo-1 cells on day −7 intravenously and treated with 1×10^6 CAR T cells intravenously on day 0. (B and E) Tumor growth was analyzed by live-cell imaging and graphed based on flux. (C and F) Kaplan-Meier survival curve. (D and G) Upon euthanasia, blood was collected and analyzed for persistence. T cells without the CAR construct (mock) were used as a control. The cell numbers of mock T cells injected in mice were based on the highest total T cell numbers within CAR T cell groups. CAR T cells were treated with vehicle or 0.25 μM capivasertib (short term or long term) before cells were injected into mice. Mann-Whitney was used for (B), (E), and (G). $n = 7$ (BAFF-R) and 8 (CD19) mice per experiment; $^*p < 0.05$, $^{**}p < 0.01$.

skewed toward memory stem (CD45RA+CD62L+), central memory (CD45RA−CD62L+), effector memory (CD45RA−CD62L−) populations and away from effector T populations (Figure 1F). Capivasertib did not impact the expression of the memory markers CD62L or CD28. There was a dose-dependent increase in the expression of the memory marker CD127 following long-term capivasertib (Figures 1G–1I and S2A–S2C). To test CAR T cell functionality following capivasertib treatment, we co-cultured CD19-CAR T cells with CD19+ Raji cells and measured interferon (IFN)-γ expression as a measurement of effector function. We observed upward trends of IFN-γ in CAR T cells treated with 0–0.5 μM capivasertib (Figure 1J), which is consistent with our previous findings.[13]

Recent evidence suggests that isolating the naive and memory T cell populations (Tn/mem) contributes to a less differentiated and more potent CAR T cell product,[8] and we have adopted this in our clinical platform.[7,9] To test how capivasertib might specifically influence Tn/mem, we manufactured Tn/mem-derived CAR T cells in 0.25 μM capivasertib (Figure 1A). Like PBMC-derived CAR T cells, we observed a decrease in the CD4+ T cells and an increase in the CD8+ T cells following both short- and long-term treatment (Figures S2A and S2B). However, Tn/mem CAR T cells treated with capivasertib retained higher levels of naive T cell populations compared to vehicle-treated Tn/mem CAR T cells (Figure S2C) as well as having increased expression of memory markers CD62L, CD127, CD28, and CD27 following both short- and long-term treatment (Figures S2D–S2I). This suggests that Tn/mem-derived CAR T cells may be more responsive to capivasertib treatment than standard PBMC-derived CAR T cells. Collectively, these data suggest that capivasertib treatment during manufacture had minimal influence on CAR T cell transduction, growth, or function but did impact the relative proportions of resulting T cell populations and phenotype. Based on these data, we selected 0.25 μM capivasertib for subsequent studies.

To test the impact of capivasertib on CAR T cell antitumor activity, we used CD19-CAR T cells that were generated with 0.25 μM capivasertib treatment (short or long term) in our mantle cell lymphoma JeKo-1 xenograft model (Figure 2A). Following treatment with mock T cells or CD19-CAR T cells, we monitored tumor burden weekly by bioluminescent imaging and performed Kaplan-Meier survival analysis. CAR T cells treated with either short- or long-term capivasertib slowed the kinetics of tumor growth compared to those treated with

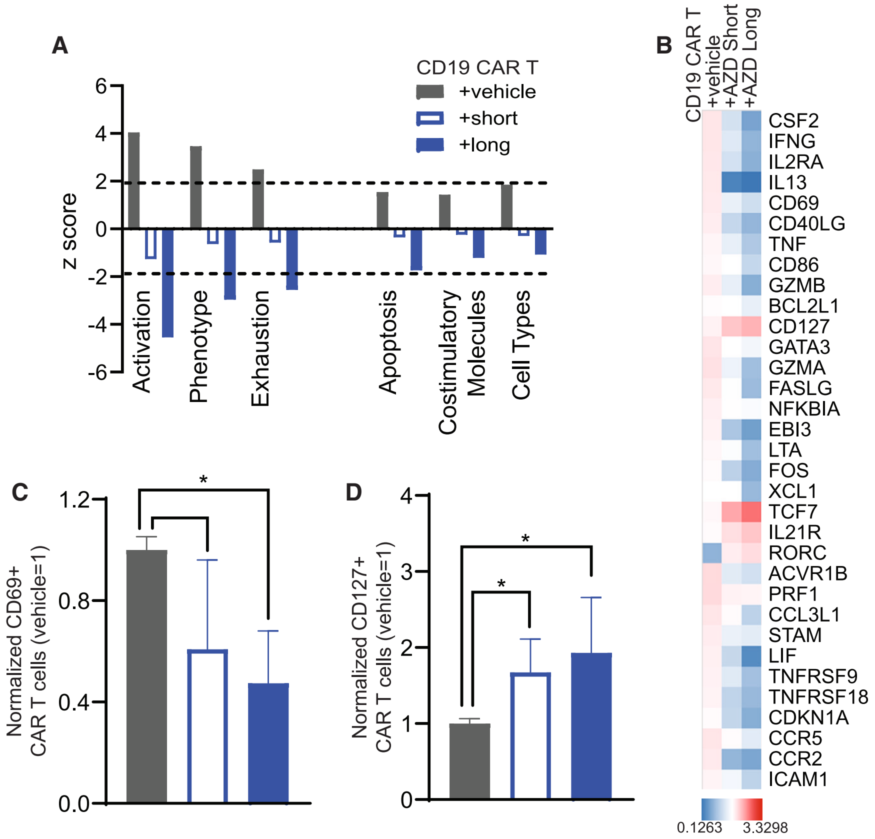

Figure 3. *Ex vivo* capivasertib treatment affected multiple genes related to CAR T cell efficacy

(A) Genes related to specific functions of T cells were analyzed through nCounter Advanced Analysis and the dash lines indicated Z score equaled 1.96 or −1.96. (B) Genes were selected based on the expression changes, which were correlated with mouse survival. (C and D) CAR T cells were cultured with DMSO or short-term/long-term capivasertib during the manufacturing process. Surface markers (CD69 and IL-7R, respectively) were detected on day 14, and data were normalized with the percentages in CAR T cells treated with vehicle. Error bar, mean ± SD; n = 4 independent experiments; *$p < 0.05$, unpaired t test.

vehicle control (Figures 2B and S3A), which translated to significantly longer mouse survival (Figure 2C). There was no difference in the activity of CAR T cells treated with short- vs. long-term capivasertib. At euthanasia, we detected CAR T cells in the blood of mice treated with cells manufactured in short- or long-term capivasertib, but not those manufactured in vehicle control (Figure 2D). We observed similar antitumor activity and prolonged survival of mice treated with Tn/mem CAR T cells manufactured with capivasertib (short or long term) (Figures S3B and S3C). However, at euthanasia, we detected CAR T cells in the blood of mice in all conditions at similar levels (Figure S3D). To confirm that capivasertib treatment would not induce non-specific antitumor efficacy of non-CAR T cells, we administered capivasertib-treated or vehicle-treated mock T cells to mice engrafted with JeKo-1 cells and observed no difference in antitumor activity or survival compared to mock T cells treated with vehicle control (Figures S3E and S3F). We subsequently tested the capivasertib-treated CAR T cells in an aggressive xenograft mouse model of Burkitt cell lymphoma Raji cells, which has a rapid growth pattern in NSG mice.[23] Even in this challenging model, CAR T cells treated with short- or long-term capivasertib induced superior survival compared to CAR T cells treated with vehicle (Figure S3G).

To determine if these observations were specific to CD19-CAR T cells, we used a CAR-targeting B cell activating factor receptor (BAFF-R)[24,25] in our JeKo-1 cell (CD19+ BAFF-R+) xenograft model (Figure 2A). Consistent with our CD19-CAR model, we observed enhanced antitumor activity of BAFF-R-CAR T cells treated with either short- or long-term capivasertib compared to vehicle control (Figure 2E), which translated into longer survival (Figure 2F). We also detected more CAR T cells in the blood of mice that received capivasertib-treated vs. vehicle control-treated BAFF-R-CAR T cells (Figure 2G). Together, these data suggest that short *ex vivo* treatment of 0.25 μM of the clinically relevant AKT inhibitor, capivasertib, enhances antitumor efficacy and prolongs survival compared to vehicle control-treated CAR T cells.

Ex vivo capivasertib exerted wide-ranging effects on CAR T cell gene expression

We performed gene expression profiling using NanoString to understand how capivasertib influences CAR T cell gene expression patterns. We focused our analysis on genes and gene pathways that could potentially explain how *ex vivo* AKT inhibition enhanced *in vivo* CAR T cell activity. Using NanoString nCounter Advanced Analysis (Figure S4A), we identified six pathways (activation, phenotype, exhaustion, apoptosis, IFN signaling, and mitogen-activated protein kinase (MAPK)/PI3K signaling) that were decreased in capivasertib-treated CAR T cells compared to vehicle control (Figure 3A). In analyzing genes within these six pathways, we observed downregulation of genes involved in T cell activation (IFN-γ [*IFNG*], *CD69*, tumor necrosis factor [*TNF*], and granzyme A and B [*GZMA* and *GZMB*]),[26,27] pro-differentiation molecules (e.g., CD40 ligand [*CD40LG*], leukemia inhibitory factor [*LIF*], and *ICAM1*),[28–30] and pro-apoptosis gene FAS ligand (*FASLG*)[31] in CAR T cells following treatment with capivasertib. Capivasertib-treated CAR T cells had increased expression of the pro-persistence genes IL-7 receptor (*IL7R*, CD127) and transcription factor 7 (*TCF7*) (Figure 3B).[32] These gene expression changes post capivasertib treatment were consistent in Tn/mem-derived CAR T cells (Figures S4B–S4D). To verify that these gene

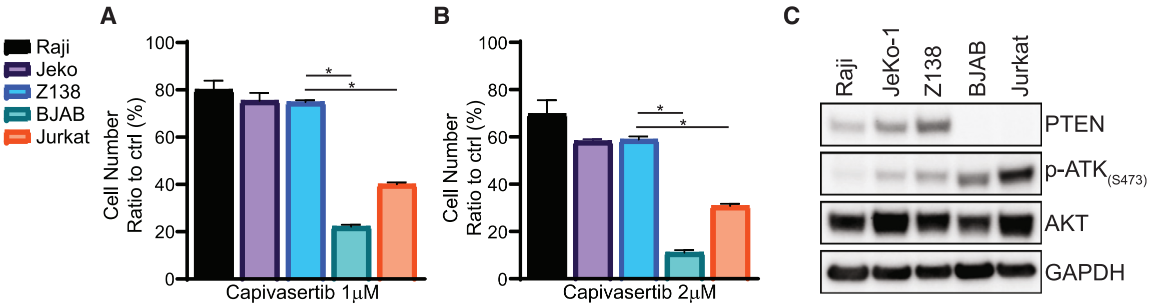

Figure 4. Tumor sensitivity to capivasertib *in vitro*

The same number of tumor cells were seeded and cultured with 1 μM (A) or 2 μM (B) capivasertib for 3 days. Cell numbers in capivasertib treatment groups were counted and compared with cell numbers in untreated groups (control). (C) Proteins were extracted from tumor cells for western blots. GAPDH was the internal control. Error bar, mean ± SD; $n = 4$ independent experiments; *$p < 0.05$, unpaired t test.

expression changes influenced protein expression, we measured the levels of CD69 and CD127 protein on CAR T cells treated with vehicle, short-, or long-term capivasertib (Figures 3C and 3D). As expected, we observed decreased CD69 and increased CD127 protein expression on CAR T cells treated with capivasertib (Figures 3C and 3D). These data suggest that both long- and short-term *ex vivo* capivasertib yield CAR T cells that are less activated and more likely to persist following infusion, which supports the enhanced *in vivo* efficacy we observed in our *in vivo* models (Figure 2).

Combination of capivasertib and CAR T cells augmented CAR T cell persistence and efficacy

Capivasertib has anti-cancer potential for tumors with *PIK3CA* or *PTEN* gene alterations[33] and is being tested in multiple in clinical trials (NCT02465060, NCT4439123, and NCT01226316) for hematological malignancies and solid tumors.[34,35] Based on our data, we hypothesized that capivasertib could be beneficial in combination with CAR T cells by exerting anti-cancer activity and potentially enhancing CAR T cell persistence. We first screened a panel of cell lines for susceptibility to capivasertib (Figures 4A and 4B) to identify cell lines that were susceptible (BJAB cells) and resistant (JeKo-1) to capivasertib to use in our experiments. We confirmed that BJAB cells lacked PTEN expression and had high levels of pAKT, while JeKo-1 cells expressed PTEN and had relatively low levels of pAKT (Figure 4C). Based on these results, we elected to use the capivasertib-resistant JeKo-1 cell line to determine the effects of capivasertib treatment on CAR T cells *in vivo* and the capivasertib-susceptible BJAB cell line to understand the combinatorial effects of CAR T cells and capivasertib in subsequent experiments.

To test the effect of capivasertib on CAR T cells *in vivo*, we administered CD19-CAR T cells into NSG mice bearing JeKo-1 tumors and concurrently treated mice with oral capivasertib (150 kg/mg) twice daily for 4 days per week over 2 weeks (Figure 5A). No obvious body weight loss was found in all groups after mice received treatment in the first 3 weeks (Figure S5A). Mice treated with CAR T cells with or without capivasertib had better tumor control (Figure 5B) and survival (Figure S5B) compared to untreated and capivasertib-only mice. However, CAR T cell engraftment was not significantly different between the groups combined with capivasertib and with vehicle (Figure 5C).

To test the potential combinatorial effects of capivasertib with CAR T cells, we repeated the previous experiment using mice engrafted intratibially with capivasertib-susceptible BJAB cells (Figure 5D). Tumor progressed slower in mice with capivasertib compared to mice with the vehicle during the 2-week capivasertib treatment period. CAR T cells with or without capivasertib decreased tumor burden compared to untreated and capivasertib alone (Figure 5E). However, the kinetics of tumor control were distinct; mice treated with CAR T cells + capivasertib had rapid control of tumor growth compared to CAR T cells + vehicle (Figure 5E). No obvious body weight loss was found in all groups after mice received treatment in the first 3 weeks (Figure S5C). We detected CAR T cells in the blood of mice treated with or without capivasertib (Figure 5F) at day 70 when all mice in these groups had undetectable tumor burden (Figure 5E). We performed a tumor rechallenge by injecting additional BJAB cells intratibially into mice that had cleared their initial tumor burden and determined that mice treated with CAR T cells without capivasertib relapsed by week 12 post rechallenge (Figure S5D). However, both groups treated with CAR T cells with or without capivasertib had superior survival compared to the mock T cell group (Figure S5E). These data suggest that combining CAR T cells with capivasertib to treat capivasertib-sensitive tumors may confer benefits over CAR T cells alone.

DISCUSSION

In vivo persistence remains a key limitation of CAR T cell therapy.[36] Although initial response rates to CAR T cells in the setting of hematological malignancies are high, durability of response remains a key area for optimization.[4] To combat the limited persistence of CAR T cells following infusion, we and others have explored a multitude of methodologies to enhance CAR T cell persistence. One such way has been to use less differentiated T cells to generate CAR T cells, which has shown positive enhancement in CAR T cell persistence.[7–9] Alternative methodologies include using membrane-bound pro-persistence cytokines allowing for autocrine signaling, however,

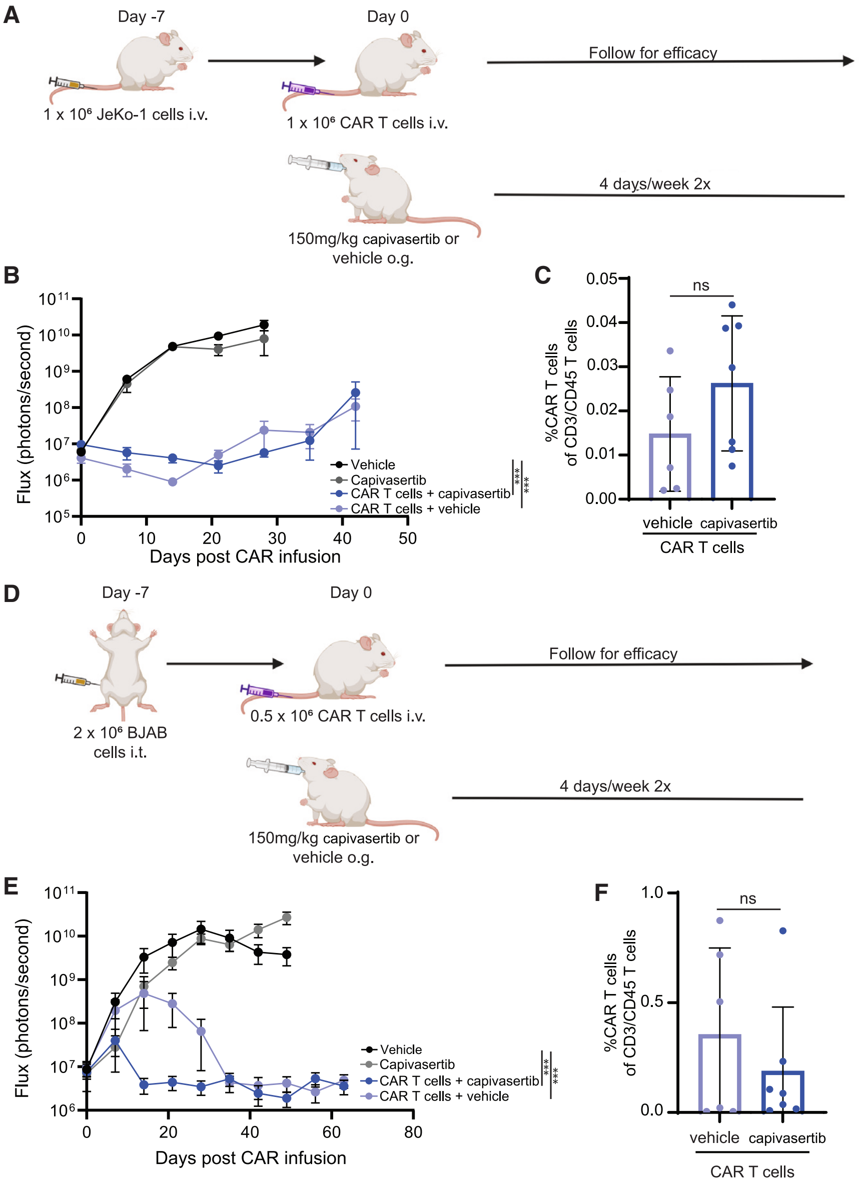

Figure 5. Combination of capivasertib and CAR T cell therapy against capivasertib-resistant and -susceptible mouse models

(A) The schema of capivasertib-resistance JeKo-1 mouse model. On day −7, mice were intravenously injected with 1×10^6 JeKo-1 cells and, on day 0, 1×10^6 CAR T cells or mock T cells were injected intravenously. Concurrently, mice were given 150 mg/kg capivasertib or vehicle (10% DMSO plus 25% Kleptose HPB buffer, the capivasertib solvent for *in vivo* mouse treatment) orally twice daily for 4 days per week over 2 weeks. (B) Tumor growth was observed by live-cell imaging and analyzed and graphed based on flux. (C) Upon euthanasia, blood was collected and analyzed for persistence. (D) The schema of capivasertib-susceptible BJAB mouse model. On day −7, mice were intratibially injected with 2×10^6 BJAB cells and, on day 0, 0.5×10^6 CAR T cells or mock T cells were injected intravenously. Concurrently, mice were given 150 mg/kg capivasertib or vehicle orally twice daily for 4 days per week over 2 weeks. (E) Tumor growth was observed by live-cell imaging and analyzed and graphed based on flux. (F) Persistence was determined by retro-orbital bleeding. T cells without the CAR construct (mock) were used as a control. The cell numbers of mock T cells injected in mice were based on the total T cell numbers in CAR T cell groups. n = 8 (mock T cell groups) and 9 (CAR T cell groups) mice per experiment in the JeKo-1 model and n = 6 and 7 (the CAR T cells + capivasertib group) mice per experiment in the BJAB model; ****$p < 0.0001$, Mann-Whitney U test.

there are concerns that this approach may increase the risk of CAR T cell transformation, potentially leading to therapy-induced diseases.[37,38] There are also methodologies to prevent CAR T cell differentiation with the AKT Inhibitor VIII, which inhibits AKT 1/2 activity.[12–14] However, we and others can not translate this into the clinic as AKT Inhibitor VIII is not clinical grade,[14] which provides a rationale to test the pan-AKT inhibitor capivasertib. Capivasertib has been US Food and Drug Administration (FDA) approved for use in breast cancer and is currently being evaluated as a single agent in hematological malignancies and solid tumors.[34,35]

We expanded on our previous work,[13] which tested the impact of adding AKT Inhibitor VIII through the course of CAR T cell culture. In this study, we optimize the use of a clinically relevant AKT inhibitor, capivasertib, for enhancing CAR T cell therapy. We specifically tested different concentrations and timing of capivasertib, including a short-term treatment, during the period of manufacturing with high levels of T cell proliferation and differentiation.[6,39–41] CAR T cells treated with short- or long-term treatment retained memory phenotypes compared to the vehicle control. Both conditions were affected by high concentrations (1 μM) of capivasertib, with the most notable effect being a significant decrease in CD4+ T cells. This is concerning as CD4 T cells have recently been shown to be a key contributor to CAR T cell efficacy.[42] However, although both long- and short-term treatments were affected by higher concentrations, long-term treatment showed reduced growth compared to short-term treatment at the same concentration, a finding consistent with observations by others.[14] Although long capivasertib treatment impacted CAR T cell manufacturing, both short- and long-treatment demonstrated equivalent activity *ex vivo* and *in vivo*. Based on these data, we conclude that treating CAR T cells during the activation period of manufacturing (i.e., short term) is the optimal strategy.

Because AKT inhibition with capivasertib during *ex vivo* manufacture enhanced CAR T cell function, we asked whether combining capivasertib with CAR T cell therapy *in vivo* would be beneficial. Capivasertib is known to have antitumor activity against *PIK3CA/AKT*-mutated tumors,[20,43,44] and therefore we tested whether this drug would enhance the efficacy of CAR T cell therapy in the context of tumor-bearing *PIK3CA/AKT* pathway mutations. Using both capivasertib-resistant and -susceptible models, we observed that capivasertib enhanced CAR T cell persistence and also augmented CAR T cell efficacy in a capivasertib-susceptible tumor model. However, further *in vivo* evaluation of tumors inherently susceptible to capivasertib is required to better assess the long-term potential of this combinatorial treatment. Collectively, our study shows that pan-AKT inhibitors have similar effects to AKT Inhibitor VIII for *ex vivo* administration and offer insight into a more optimized regiment for both PBMC- and Tn/mem-derived CAR T cells in clinical manufacturing to favor a memory phenotype without changing the process of CAR T cell manufacturing. Furthermore, since capivasertib has antitumor effects on tumors with *PTEN*-mutations,[33] our study shows a new potential combinatorial strategy for CAR T cells in hematological and solid tumors.

MATERIALS AND METHODS

Blood cell isolation, CAR T cell generation, and cell maintenance

Human blood was obtained from the City of Hope Blood Donor Apheresis Center under protocols approved by the Institutional Review Board (IRB). Peripheral blood mononuclear cells (PBMCs) were isolated by Ficoll-Paque PLUS density gradient medium (17144002, Cytiva) following the manufacturer's instructions. To obtain naive and stem cell memory T cell (Tn/mem) populations, PBMCs were incubated with CD14 and CD25 microbeads to eliminate monocytes and regulatory T cells, respectively, followed by CD62L microbeads to enrich Tn/mem using autoMACS Pro Separator (Miltenyi Biotech).

To generate CAR T cells, 1×10^6 PBMCs or Tn/mem were transduced with a lentivirus to express our CD19-directed CAR construct[13] or BAFF-R-directed CAR construct,[24] as described.[13] PBMC-or Tn/mem-derived CAR T cells were stimulated with Human T-expander CD3/CD28 Dynabeads (Gibco) (Ccells:Dynabeads = 1:3) for 7 days, followed by bead removal. CAR T cells were expanded in RPMI 1640 medium containing 10% fetal bovine serum (FBS; SH30070.03HI, HyClone), 50 U/mL recombinant human IL-2 (rhIL-2, NDC0078-0495-61, Novartis), 0.5 ng/mL recombinant human IL-15 (rhIL-15, 1013-050, CellGenix), and indicated concentrations of capivasertib (AZD5363, Trucap, AstraZeneca) dissolved in DMSO (vehicle) for 16 days. Medium was supplemented with rhIL-2, rhIL-15, and capivasertib/vehicle every other day without removing existing reagents and medium. Capivasertib was diluted in the vehicle to 0.25, 0.5, and 1 mM from the stock solution (2 mM in the vehicle) before adding it into each cell culture vessel to ensure the final concentration of the vehicle was the same in each group, including the group treated with 0 μM capivasertib (vehicle alone). In each experimental group, the same numbers of cells were seeded with CD3/CD28 Dynabeads and then cells were expanded based on cell concentration every 2 days without removing any cells until day 16. Cell numbers and viability were measured every other day using a Guava Muse Cell Analyzer (Cytek).

Raji, Z-138, BJAB (Thermo Fisher Scientific), LCL, KG-1a, and Jurkat cell lines were cultured in RPMI 1640 medium (BE12-115F, Lonza) with 10% FBS. JeKo-1 cells were maintained in RPMI 1640 medium with 20% FBS. LCL cells over-expressing the human anti-CD3 antibody, OKT3 (LCL-OKT3), were cultured in RPMI 1640 medium with 10% FBS and hygromycin B (InvivoGen, ant-hg-1). All cells including CAR T cells were cultured at 37°C and 5% carbon dioxide.

Antibodies and reagents

The following antibodies were used for western blots: anti-GSK-3β (12456S, Cell Signaling), anti-phospho-GSK-3β (S9, 5558S, Cell Signaling), anti-AKT (4691S, Cell Signaling), anti-phospho-AKT (S473, 4060S, Cell Signaling), anti-PTEN (9559S, Cell Signaling), anti-β-actin (3700S, Cell Signaling), anti-GAPDH (sc-32233, Santa Cruz), goat anti-rabbit IgG (HRP, ab205718, Abcam), and goat anti-mouse IgG (HRP, ab205719, Abcam) antibodies. Amersham ECL Western Blotting Detection Reagents (RPN 2209, Cytiva) and ChemiDoc MP Imaging System (Bio-Rad) were used for chemiluminescent signaling detection.

Cells were stained with fluorochrome-conjugated antibodies against the following antigens for flow cytometry: anti-CD3 (563109), anti-CD4 (340443, 340133), anti-CD8 (348793), anti-CD27 (555440, 555441), anti-CD28 (561368, 555728), anti-CD45 (340665), anti-CD45RA (555489), anti-CD45RO (561137), anti-CD62L (559772, 341012), anti-CD127 (557938), and anti-IFN-γ (554702) from BD Biosciences; anti-EGFR (352906, 352904, 352910) and anti-KLRG1 (138408, 138416) from BioLegend; anti-TIM3 (17-3109-42, 25-3109-42) and anti-PD-1 (11-9969, 12-9969-42) from eBioscience, and anti-CD3 (130-113-133) from Miltenyi Biotech. Cells were stained with 4′,6-diamidino-2-phenylindole (DAPI, D21490, Invitrogen) or fixable viability dye (FVD, 65-0866-18, eBioscience) to distinguish live cells from dead cells. For intracellular staining, cells were fixed and permeabilized with Cytofix/Cytoperm Fixation/Permeabilization Solution Kit (554714, BD Bioscience) before staining. To enhance the detectability of cytokines by intracellular staining, GolgiPlug protein transport inhibitor containing Brefeldin A (555029, BD Bioscience) was added following the manufacturer's protocol. All flow cytometry was performed using MACSQuant Analyzer 10 (Miltenyi Biotech) and analyzed using FCS Express version 7 (De Novo Software).

Cytokine production assay

CAR T cells were co-cultured with a Raji cell line at a 1:1 ratio at 37°C for 4 h before adding Brefeldin A GolgiPlug (BD Bioscience) and then

incubated for 24 h at 37°C before intracellular staining for IFN-γ as described above.

NanoString gene expression analysis

Total RNA from T cells was purified following sample requirements for the NanoString Gene Expression Assay and nCounter CAR-T Characterization Panel (NanoString Technologies). Raw data were normalized and processed with nSolver 4.0 Analysis software and nCounter Advanced Analysis 2.0 software following manufacturer's instructions (NanoString Technologies).

Xenograft models

All models included tumor cells expressing firefly luciferase and enhanced green fluorescent protein (ffLuc-eGFP) fusion proteins, mock T or CAR T cells derived from PBMCs or Tn/mem cells, capivasertib dissolved in 10% DMSO vehicle plus 25% (w/v) Kleptose HPB buffer (Roquette), and 6- to 8-week-old male NOD-*Scid*IL2Rγ^{null} (NSG) mice. Tumor cells (0.5 $\times$ 10^6 [Raji; intravenous], 1 $\times$ 10^6 [JeKo-1; intravenous], or 2 $\times$ 10^6 [BJAB; intratibial]) were injected into mice and allowed to engraft until tumor signal was detected by bioluminescence imaging. The treatment included intravenous injection of mock T/CAR T cells (the total amount of the cells was based on 1 or 0.5 $\times$ 10^6 EGFR+ T cells in the CAR T cell group) alone or plus oral vehicle/capivasertib (150 mg/kg twice daily for 4 days per week over 2 weeks).[21] Mouse body weight, appearance, and activity were monitored at least once a week. The experimental humane endpoint was determined by body weight loss (loss of 15%–20% within a few days or consistently gradual weight loss), poor external physical appearance (such as severe hunching and the presence of labored respiration), and stationary activity until stimulation. Tumor burden was measured and analyzed by intraperitoneal injection of D-luciferin for bioluminescence imaging using SPECTRAL Lago X imaging system (Spectral Instruments Imaging) and Aura Imaging Software (Spectral Instruments Imaging) once a week. Retro-orbital blood collection was performed for T cell analysis. Peripheral blood was collected after euthanasia. Animal experiments were performed under protocols approved by the City of Hope's Institutional Animal Care and Use Committee (IACUC).

Statistics

Unpaired t test, Mann-Whitney U test, and Kaplan-Meier methods were used to analyze *in vitro* and *in vivo* experiments using GraphPad Prism 9 (GraphPad Software). A p value $\leq$0.05 was considered statistically significant.

DATA AND CODE AVAILABILITY

For original data, please contact the corresponding author.

ACKNOWLEDGMENTS

The authors thank the Leukemia and Lymphoma Society Mantle Cell Lymphoma Research Initiative (SCOR 7000-18; PI, L.W.K.; Project Leaders: S.J.F. and X.W.) for funding and AstraZeneca for providing capivasertib for the study.

AUTHOR CONTRIBUTIONS

X.W., L.W.K., and S.J.F. contributed to the study concept and design and data interpretation. H.-J.H. performed experiments and generated figures. X.W., R.U., H.-J.H., and M.C.C. wrote the manuscript.

DECLARATION OF INTERESTS

The authors do declare no competing interests.

SUPPLEMENTAL INFORMATION

Supplemental information can be found online at https://doi.org/10.1016/j.omtm.2025.101421.

REFERENCES

1. Neelapu, S.S., Locke, F.L., Bartlett, N.L., Lekakis, L.J., Miklos, D.B., Jacobson, C.A., Braunschweig, I., Oluwole, O.O., Siddiqi, T., Lin, Y., et al. (2017). Axicabtagene Ciloleucel CAR T-Cell Therapy in Refractory Large B-Cell Lymphoma. N. Engl. J. Med. *377*, 2531–2544. https://doi.org/10.1056/NEJMoa1707447.

2. Raje, N., Berdeja, J., Lin, Y., Siegel, D., Jagannath, S., Madduri, D., Liedtke, M., Rosenblatt, J., Maus, M.V., Turka, A., et al. (2019). Anti-BCMA CAR T-Cell Therapy bb2121 in Relapsed or Refractory Multiple Myeloma. N. Engl. J. Med. *380*, 1726–1737. https://doi.org/10.1056/NEJMoa1817226.

3. Schuster, S.J., Svoboda, J., Chong, E.A., Nasta, S.D., Mato, A.R., Anak, Ö., Brogdon, J.L., Pruteanu-Malinici, I., Bhoj, V., Landsburg, D., et al. (2017). Chimeric Antigen Receptor T Cells in Refractory B-Cell Lymphomas. N. Engl. J. Med. *377*, 2545–2554. https://doi.org/10.1056/NEJMoa1708566.

4. Cappell, K.M., Sherry, R.M., Yang, J.C., Goff, S.L., Vanasse, D.A., McIntyre, L., Rosenberg, S.A., and Kochenderfer, J.N. (2020). Long-Term Follow-Up of Anti-CD19 Chimeric Antigen Receptor T-Cell Therapy. J. Clin. Oncol. *38*, 3805–3815. https://doi.org/10.1200/JCO.20.01467.

5. Bar, N., Diels, J., van Sanden, S., Mendes, J., Hernando, T., Cost, P., Schecter, J.M., Lendvai, N., Patel, N., Ishida, T., et al. (2023). Comparative Efficacy of Ciltacabtagene Autoleucel Versus Idecabtagene Vicleucel in the Treatment of Patients with Relapsed or Refractory Multiple Myeloma Previously Treated with 2-4 Prior Lines of Therapy Using a Matching-Adjusted Indirect Comparison. Blood *142*, 2141. https://doi.org/10.1182/blood-2023-182141.

6. Alizadeh, D., Wong, R.A., Yang, X., Wang, D., Pecoraro, J.R., Kuo, C.F., Aguilar, B., Qi, Y., Ann, D.K., Starr, R., et al. (2019). IL15 Enhances CAR-T Cell Antitumor Activity by Reducing mTORC1 Activity and Preserving Their Stem Cell Memory Phenotype. Cancer Immunol. Res. *7*, 759–772. https://doi.org/10.1158/2326-6066.CIR-18-0466.

7. Aldoss, I., Khaled, S.K., Wang, X., Palmer, J., Wang, Y., Wagner, J.R., Clark, M.C., Simpson, J., Paul, J., Vyas, V., et al. (2023). Favorable Activity and Safety Profile of Memory-Enriched CD19-Targeted Chimeric Antigen Receptor T-Cell Therapy in Adults with High-Risk Relapsed/Refractory ALL. Clin. Cancer Res. *29*, 742–753. https://doi.org/10.1158/1078-0432.ccr-22-2038.

8. Arcangeli, S., Bove, C., Mezzanotte, C., Camisa, B., Falcone, L., Manfredi, F., Bezzecchi, E., El Khoury, R., Norata, R., Sanvito, F., et al. (2022). CAR T cell manufacturing from naive/stem memory T lymphocytes enhances antitumor responses while curtailing cytokine release syndrome. J. Clin. Invest. *132*, e150807. https://doi.org/10.1172/jci150807.

9. Brown, C.E., Alizadeh, D., Starr, R., Weng, L., Wagner, J.R., Naranjo, A., Ostberg, J.R., Blanchard, M.S., Kilpatrick, J., Simpson, J., et al. (2016). Regression of Glioblastoma after Chimeric Antigen Receptor T-Cell Therapy. N. Engl. J. Med. *375*, 2561–2569. https://doi.org/10.1056/NEJMoa1610497.

10. Jafarzadeh, L., Masoumi, E., Mirzaei, H.R., Alishah, K., Fallah-Mehrjardi, K., Khakpoor-Koosheh, M., Rostamian, H., Noorbakhsh, F., and Hadjati, J. (2021). Targeted knockdown of Tim3 by short hairpin RNAs improves the function of anti-mesothelin CAR T cells. Mol. Immunol. *139*, 1–9. https://doi.org/10.1016/j.molimm.2021.06.007.

11. Rupp, L.J., Schumann, K., Roybal, K.T., Gate, R.E., Ye, C.J., Lim, W.A., and Marson, A. (2017). CRISPR/Cas9-mediated PD-1 disruption enhances anti-tumor efficacy of

human chimeric antigen receptor T cells. Sci. Rep. *7*, 737. https://doi.org/10.1038/s41598-017-00462-8.

12. Klebanoff, C.A., Crompton, J.G., Leonardi, A.J., Yamamoto, T.N., Chandran, S.S., Eil, R.L., Sukumar, M., Vodnala, S.K., Hu, J., Ji, Y., et al. (2017). Inhibition of AKT signaling uncouples T cell differentiation from expansion for receptor-engineered adoptive immunotherapy. JCI Insight *2*, e95103. https://doi.org/10.1172/jci.insight.95103.
13. Urak, R., Walter, M., Lim, L., Wong, C.W., Budde, L.E., Thomas, S., Forman, S.J., and Wang, X. (2017). Ex vivo Akt inhibition promotes the generation of potent CD19CAR T cells for adoptive immunotherapy. J. Immunother. Cancer *5*, 26. https://doi.org/10.1186/s40425-017-0227-4.
14. Vedika, M., Giulia, A., Juliana Dias Alves, P., Manar, S.S., Amaia Cadinanos, G., Louisa, G., Alastair, H., Fred Arce, V., Karl, S.P., Anniek, B.v.d.W., et al. (2023). AKT inhibition generates potent polyfunctional clinical grade AUTO1 CAR T-cells, enhancing function and survival. J. Immunother. Cancer *11*, e007002. https://doi.org/10.1136/jitc-2023-007002.
15. Kim, E.H., and Suresh, M. (2013). Role of PI3K/Akt signaling in memory CD8 T cell differentiation. Front. Immunol. *4*, 20. https://doi.org/10.3389/fimmu.2013.00020.
16. Bhattacharyya, N.D., and Feng, C.G. (2020). Regulation of T Helper Cell Fate by TCR Signal Strength. Front. Immunol. *11*, 624. https://doi.org/10.3389/fimmu.2020.00624.
17. Nitulescu, G.M., Van De Venter, M., Nitulescu, G., Ungurianu, A., Juzenas, P., Peng, Q., Olaru, O.T., Grădinaru, D., Tsatsakis, A., Tsoukalas, D., et al. (2018). The Akt pathway in oncology therapy and beyond (Review). Int. J. Oncol. *53*, 2319–2331. https://doi.org/10.3892/ijo.2018.4597.
18. Han, J.M., Patterson, S.J., and Levings, M.K. (2012). The Role of the PI3K Signaling Pathway in CD4(+) T Cell Differentiation and Function. Front. Immunol. *3*, 245. https://doi.org/10.3389/fimmu.2012.00245.
19. Zhang, Q., Ding, J., Sun, S., Liu, H., Lu, M., Wei, X., Gao, X., Zhang, X., Fu, Q., and Zheng, J. (2019). Akt inhibition at the initial stage of CAR-T preparation enhances the CAR-positive expression rate, memory phenotype and in vivo efficacy. Am. J. Cancer Res. *9*, 2379–2396.
20. Nitulescu, G.M., Margina, D., Juzenas, P., Peng, Q., Olaru, O.T., Saloustros, E., Fenga, C., Spandidos, D.A., Libra, M., and Tsatsakis, A.M. (2016). Akt inhibitors in cancer treatment: The long journey from drug discovery to clinical use (Review). Int. J. Oncol. *48*, 869–885. https://doi.org/10.3892/ijo.2015.3306.
21. Davies, B.R., Greenwood, H., Dudley, P., Crafter, C., Yu, D.H., Zhang, J., Li, J., Gao, B., Ji, Q., Maynard, J., et al. (2012). Preclinical pharmacology of AZD5363, an inhibitor of AKT: pharmacodynamics, antitumor activity, and correlation of monotherapy activity with genetic background. Mol. Cancer Ther. *11*, 873–887. https://doi.org/10.1158/1535-7163.MCT-11-0824-T.
22. Okuzumi, T., Fiedler, D., Zhang, C., Gray, D.C., Aizenstein, B., Hoffman, R., and Shokat, K.M. (2009). Inhibitor hijacking of Akt activation. Nat. Chem. Biol. *5*, 484–493. https://doi.org/10.1038/nchembio.183.
23. Xiao, Q., and Su, X. (2023). Anti-tumor Efficacy of CD19 CAR-T in a Raji B Cell Xenografted Mouse Model. Bio. Protoc. *13*, e4655. https://doi.org/10.21769/BioProtoc.4655.
24. Qin, H., Dong, Z., Wang, X., Cheng, W.A., Wen, F., Xue, W., Sun, H., Walter, M., Wei, G., Smith, D.L., et al. (2019). CAR T cells targeting BAFF-R can overcome CD19 antigen loss in B cell malignancies. Sci. Transl. Med. *11*, eaaw9414. https://doi.org/10.1126/scitranslmed.aaw9414.
25. Wang, X., Dong, Z., Awuah, D., Chang, W.C., Cheng, W.A., Vyas, V., Cha, S.C., Anderson, A.J., Zhang, T., Wang, Z., et al. (2022). CD19/BAFF-R dual-targeted CAR T cells for the treatment of mixed antigen-negative variants of acute lymphoblastic leukemia. Leukemia *36*, 1015–1024. https://doi.org/10.1038/s41375-021-01477-x.
26. Grossman, W.J., Verbsky, J.W., Tollefsen, B.L., Kemper, C., Atkinson, J.P., and Ley, T.J. (2004). Differential expression of granzymes A and B in human cytotoxic lymphocyte subsets and T regulatory cells. Blood *104*, 2840–2848. https://doi.org/10.1182/blood-2004-03-0859.
27. Mita, Y., Kimura, M.Y., Hayashizaki, K., Koyama-Nasu, R., Ito, T., Motohashi, S., Okamoto, Y., and Nakayama, T. (2018). Crucial role of CD69 in anti-tumor immunity through regulating the exhaustion of tumor-infiltrating T cells. Int. Immunol. *30*, 559–567. https://doi.org/10.1093/intimm/dxy050.
28. Tay, N.Q., Lee, D.C.P., Chua, Y.L., Prabhu, N., Gascoigne, N.R.J., and Kemeny, D.M. (2017). CD40L Expression Allows CD8+ T Cells to Promote Their Own Expansion and Differentiation through Dendritic Cells. Front. Immunol. *8*, 1484. https://doi.org/10.3389/fimmu.2017.01484.
29. Metcalfe, S.M. (2011). LIF in the regulation of T-cell fate and as a potential therapeutic. Genes Immun. *12*, 157–168. https://doi.org/10.1038/gene.2011.9.
30. Cox, M.A., Barnum, S.R., Bullard, D.C., and Zajac, A.J. (2013). ICAM-1–dependent tuning of memory CD8 T-cell responses following acute infection. Proc. Natl. Acad. Sci. USA *110*, 1416–1421. https://doi.org/10.1073/pnas.1213480110.
31. Zeytun, A., Hassuneh, M., Nagarkatti, M., and Nagarkatti, P.S. (1997). Fas-Fas Ligand–Based Interactions Between Tumor Cells and Tumor-Specific Cytotoxic T Lymphocytes: A Lethal Two-Way Street. Blood *90*, 1952–1959. https://doi.org/10.1182/blood.V90.5.1952.
32. Chen, G.M., Chen, C., Das, R.K., Gao, P., Chen, C.H., Bandyopadhyay, S., Ding, Y.Y., Uzun, Y., Yu, W., Zhu, Q., et al. (2021). Integrative Bulk and Single-Cell Profiling of Premanufacture T-cell Populations Reveals Factors Mediating Long-Term Persistence of CAR T-cell Therapy. Cancer Discov. *11*, 2186–2199. https://doi.org/10.1158/2159-8290.cd-20-1677.
33. Smyth, L.M., Batist, G., Meric-Bernstam, F., Kabos, P., Spanggaard, I., Lluch, A., Jhaveri, K., Varga, A., Wong, A., Schram, A.M., et al. (2021). Selective AKT kinase inhibitor capivasertib in combination with fulvestrant in PTEN-mutant ER-positive metastatic breast cancer. NPJ Breast Cancer *7*, 44. https://doi.org/10.1038/s41523-021-00251-7.
34. Turner, N.C., Oliveira, M., Howell, S.J., Dalenc, F., Cortes, J., Gomez Moreno, H.L., Hu, X., Jhaveri, K., Krivorotko, P., Loibl, S., et al. (2023). Capivasertib in Hormone Receptor–Positive Advanced Breast Cancer. N. Engl. J. Med. *388*, 2058–2070. https://doi.org/10.1056/NEJMoa2214131.
35. Wang, M., Shin, H.-J., Herrera, A.F., Levy, M., Kim, W.S., Kim, T.M., Kim, J.S., Yoon, D.H., Ribrag, V., Canales, M., et al. (2022). Abstract CT127: Trial in progress: A phase II, open-label, multicenter study of capivasertib, a potent, oral pan-AKT inhibitor in patients with relapsed or refractory B-cell non-hodgkin lymphoma (CAPITAL). Cancer Res. *82*, CT127. https://doi.org/10.1158/1538-7445.am2022-ct127.
36. Xu, X., Sun, Q., Liang, X., Chen, Z., Zhang, X., Zhou, X., Li, M., Tu, H., Liu, Y., Tu, S., and Li, Y. (2019). Mechanisms of Relapse After CD19 CAR T-Cell Therapy for Acute Lymphoblastic Leukemia and Its Prevention and Treatment Strategies. Front. Immunol. *10*, 2664. https://doi.org/10.3389/fimmu.2019.02664.
37. Jun Lee, E.H., Cullen, C., Murad, J.P., Gumber, D., Park, A.K., Yang, J., Stern, L.A., Adkins, L.N., Dhapola, G., Gittins, B., et al. (2023). Antigen-dependent IL-12 signaling in CAR T cells promotes regional to systemic disease targeting. Preprint at bioRxiv. https://doi.org/10.1101/2023.01.06.522784.
38. Shum, T., Omer, B., Tashiro, H., Kruse, R.L., Wagner, D.L., Parikh, K., Yi, Z., Sauer, T., Liu, D., Parihar, R., et al. (2017). Constitutive Signaling from an Engineered IL7 Receptor Promotes Durable Tumor Elimination by Tumor-Redirected T Cells. Cancer Discov. *7*, 1238–1247. https://doi.org/10.1158/2159-8290.cd-17-0538.
39. Hoffmann, J.M., Schubert, M.L., Wang, L., Hückelhoven, A., Sellner, L., Stock, S., Schmitt, A., Kleist, C., Gern, U., Loskog, A., et al. (2017). Differences in Expansion Potential of Naive Chimeric Antigen Receptor T Cells from Healthy Donors and Untreated Chronic Lymphocytic Leukemia Patients. Front. Immunol. *8*, 1956. https://doi.org/10.3389/fimmu.2017.01956.
40. Mitchell, D.M., Ravkov, E.V., and Williams, M.A. (2010). Distinct roles for IL-2 and IL-15 in the differentiation and survival of CD8+ effector and memory T cells. J. Immunol. *184*, 6719–6730. https://doi.org/10.4049/jimmunol.0904089.
41. Klebanoff, C.A., Finkelstein, S.E., Surman, D.R., Lichtman, M.K., Gattinoni, L., Theoret, M.R., Grewal, N., Spiess, P.J., Antony, P.A., Palmer, D.C., et al. (2004). IL-15 enhances the in vivo antitumor activity of tumor-reactive CD8+ T cells. Proc. Nat. Acad. Sci. USA *101*, 1969–1974. https://doi.org/10.1073/pnas.0307298101.

42. Wang, D., Aguilar, B., Starr, R., Alizadeh, D., Brito, A., Sarkissian, A., Ostberg, J.R., Forman, S.J., and Brown, C.E. (2018). Glioblastoma-targeted CD4+ CAR T cells mediate superior antitumor activity. JCI Insight *3*, e99048. https://doi.org/10.1172/jci.insight.99048.

43. Willis, B., Neveras, I., Dry, H., Xu, W., Li, Y., Mongeon, K., Roderick, J., Rosen, A., Klener, P., Lenz, G., et al. (2021). Combination Benefit of Capivasertib and Venetoclax in Preclinical Models of Diffuse Large B-Cell Lymphoma. Blood *138*, 1870.

44. Fusco, N., Sajjadi, E., Venetis, K., Gaudioso, G., Lopez, G., Corti, C., Rocco, E.G., Criscitiello, C., Malapelle, U., and Invernizzi, M. (2020). PTEN Alterations and Their Role in Cancer Management: Are We Making Headway on Precision Medicine? Genes *11*, 719. https://doi.org/10.3390/genes11070719.

OMTM, Volume *33*

Supplemental information

Capivasertib enhances chimeric antigen receptor T cell activity in preclinical models of B cell lymphoma

Hui-Ju Hsieh, Ryan Urak, Mary C. Clark, Larry W. Kwak, Stephen J. Forman, and Xiuli Wang

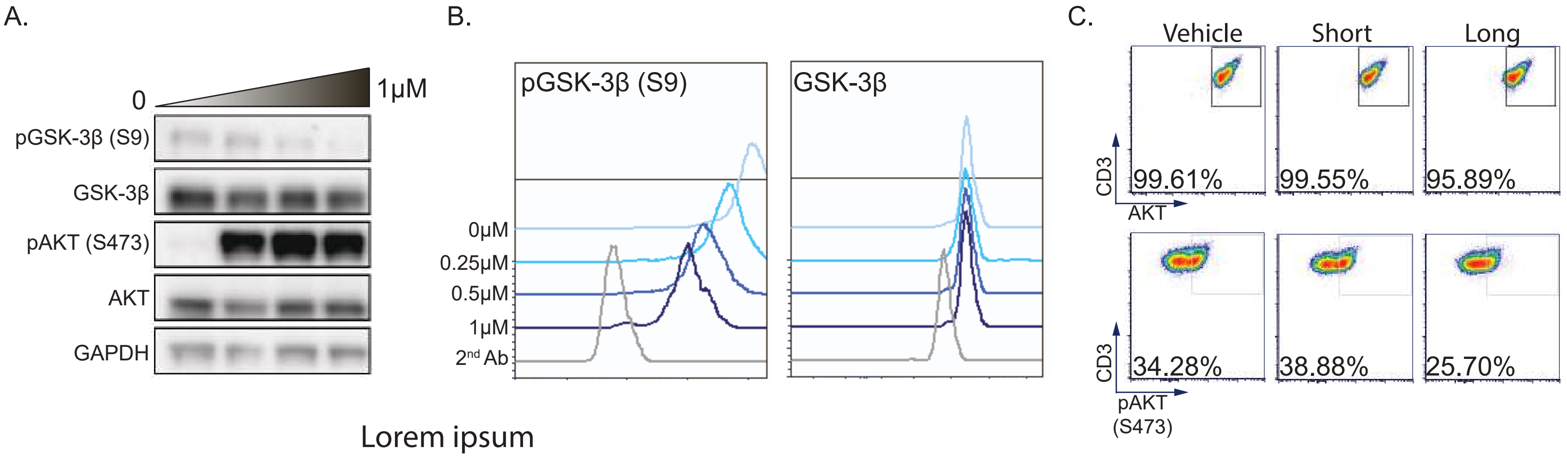

Figure S1. AKT signaling in CAR T cells was regulated by ex vivo capivasertib treatment.(A-B) CD19 CAR T cells were expanded for 14 days and treated with various concentrations of capivasertib for 2 hours: (A) PBMC-derived (0, 0.25, 0.5, 1, and 2 μM) and (B) Tn/mem-derived (0, 0.25, 0.5, and 1 μM). Westernblot and flow cytometry analyses were performed immediately after capivasertib treatment. GAPDH served as the loading control for Western blots, while cells stained only with the secondary antibody were used as flow cytometry controls. (C) The expressions of AKT and pAKT (S473) were evaluated following treatment of CD19 CAR T cells with 0.25 μM capivasertib under short-term and long-term conditions. Control (Ctrl) cells were treated with the same volume of DMSO as the long-term capivasertib-treated group. Data represent n = 4 independent experiments, and error bars denote mean ± SD.

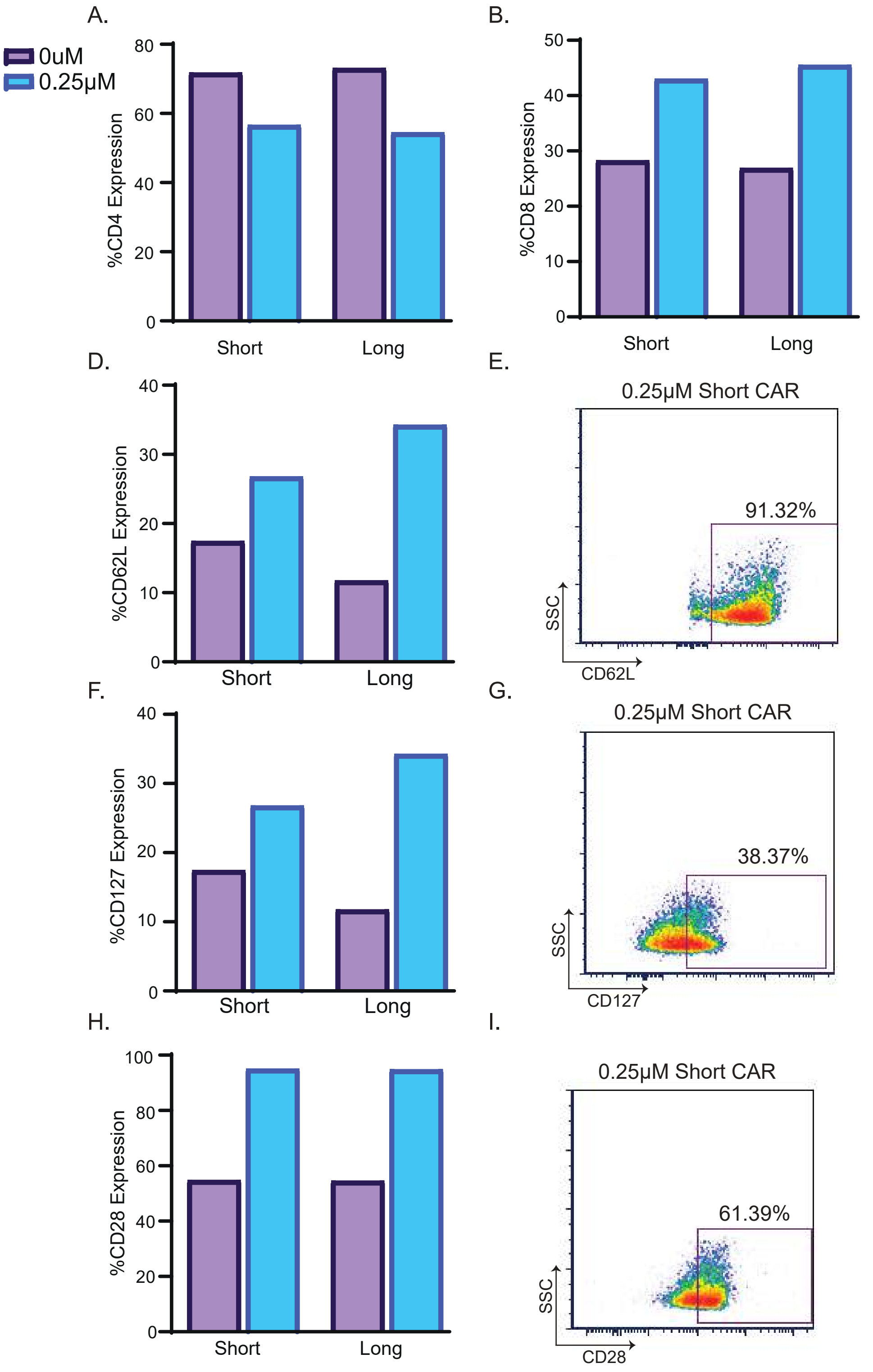

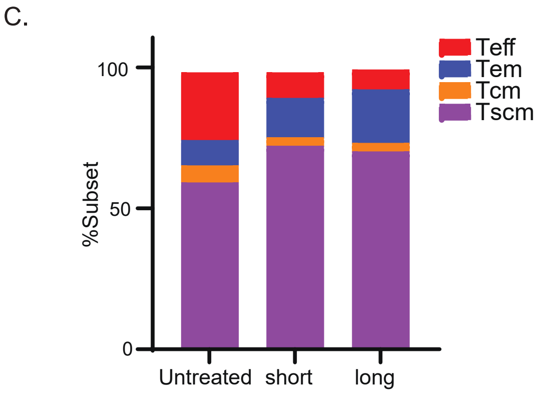

Figure S2. Ex vivo capivasertib treatment on less differentiated T cells showed phenotypic changes. The same amounts of cells with Dynabeads were seeded on Day 0 in each group. Dynabeads were removed on Day 7. Cells were expanded by adding fresh media with final concentrations of 50 U/mL of rhIL-2, 0.5 ng/mL of rhIL-15, and capivasertib (0.25μM) and vehicle (0μ M) every two days until Day 16. Percent expression of CD4+(A), CD8+(B), T cell subsets (C), and memory markers, CD62L (D-E), CD127 (F-G), CD28 (H-I) on short and long capivasertib treated CAR T cells (CAR gated) at Day 14. n = 4 independent experiments, error bars: mean ± SD, * $p<0.05$, ** $p<0.01$ unpaired t-test. T effector cells (Teff), T effector memory cells (Tem), T central memory cells (Tcm), and T memory stem cells (Tscm).

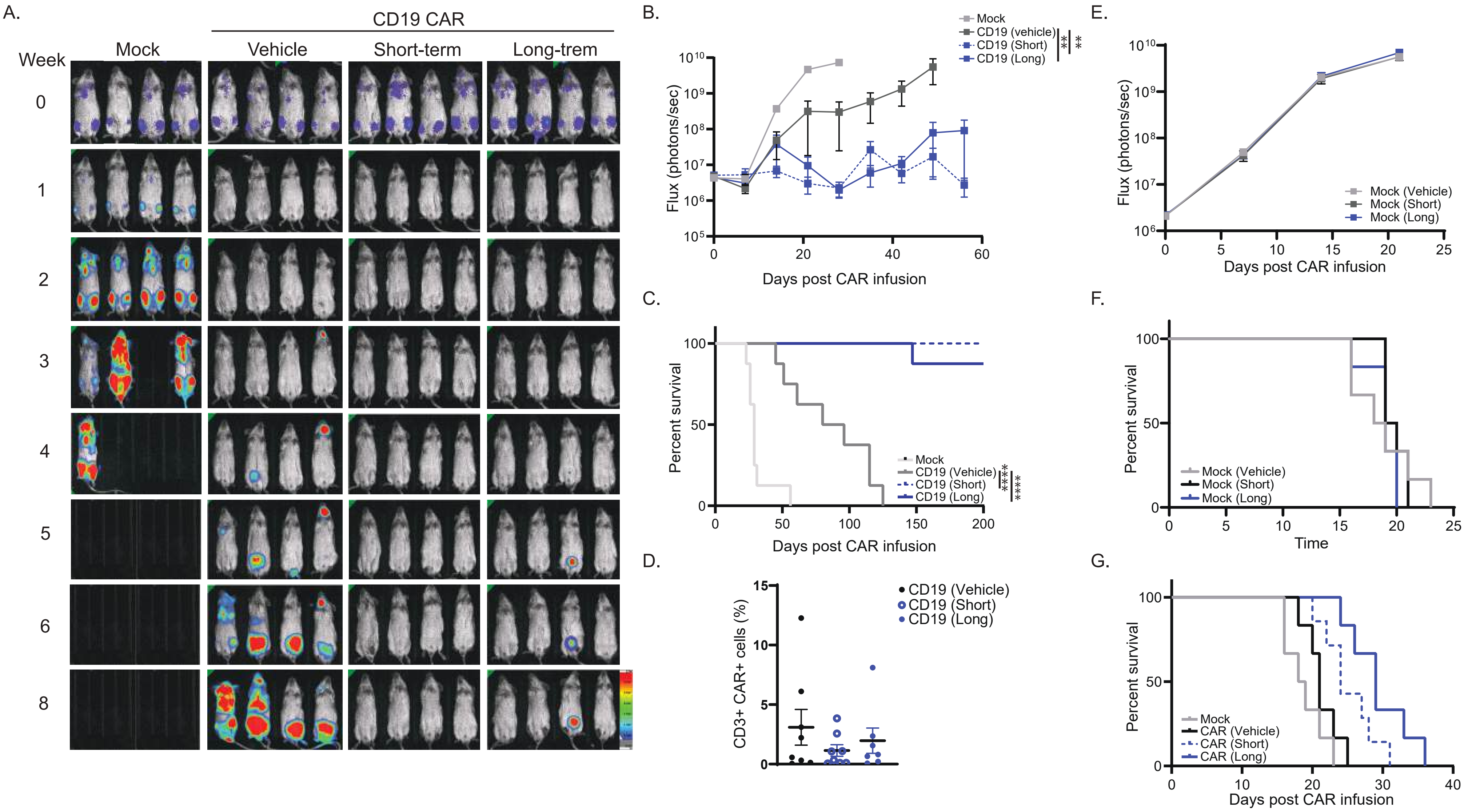

Figure S3. Ex vivo capivasertib treatment enhances CAR T cells efficacy in vivo 1 × 10^6 JeKo-1 cells were injected intravenously into NSG mice at day -7, followed by treatment with 1 × 10^6 CAR T cells administered intravenously. (A-B) Tumor growth was monitored using live mouse imaging and quantified based on bioluminescent flux. (C) Kaplan-Meier survival curve analysis. (D) Blood samples were collected upon euthanasia to assess CAR T cell persistence. 0.5 × 10^6 Raji cells were injected intravenously at day -7, and mice were treated with Mock cells consistent with the total amount of T cells in CAR treated experiments intravenously. (E) Tumor growth was analyzed via live mouse imaging and graphed based on flux. (F-G) Kaplan-Meier survival curve analysis was performed. Mock cells were used as a control. Statistical analysis was conducted using the Mann-Whitney test (** p<0.01, **** p < 0.0001).

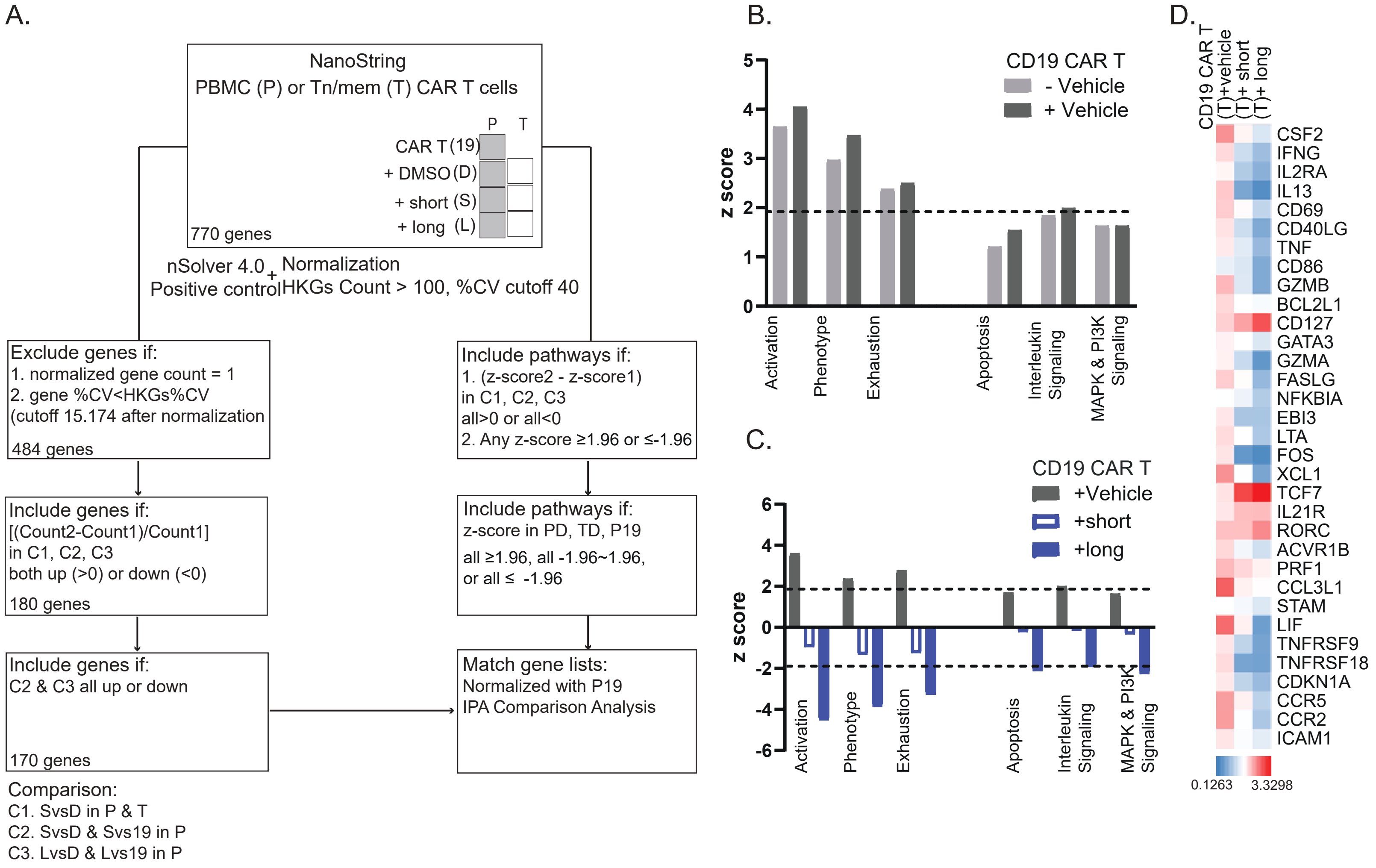

Figure S4: Ex vivo capivasertib treatment regulated multiple functions of CAR T cells
(A) The flowchart for NanoString gene expression data analysis. (B) Genes related to specific functions of T cells were analyzed and the dash line indicated z score equaled 1.96. (C) Genes related to specific functions of Tn/mem CAR T cells were analyzed through nCounter Advanced Analysis and the dash lines indicated z-score equaled 1.96 or -1.96. (D) Genes were selected based on the expression changes which were correlated with mouse survival.; n = 4 independent experiments.

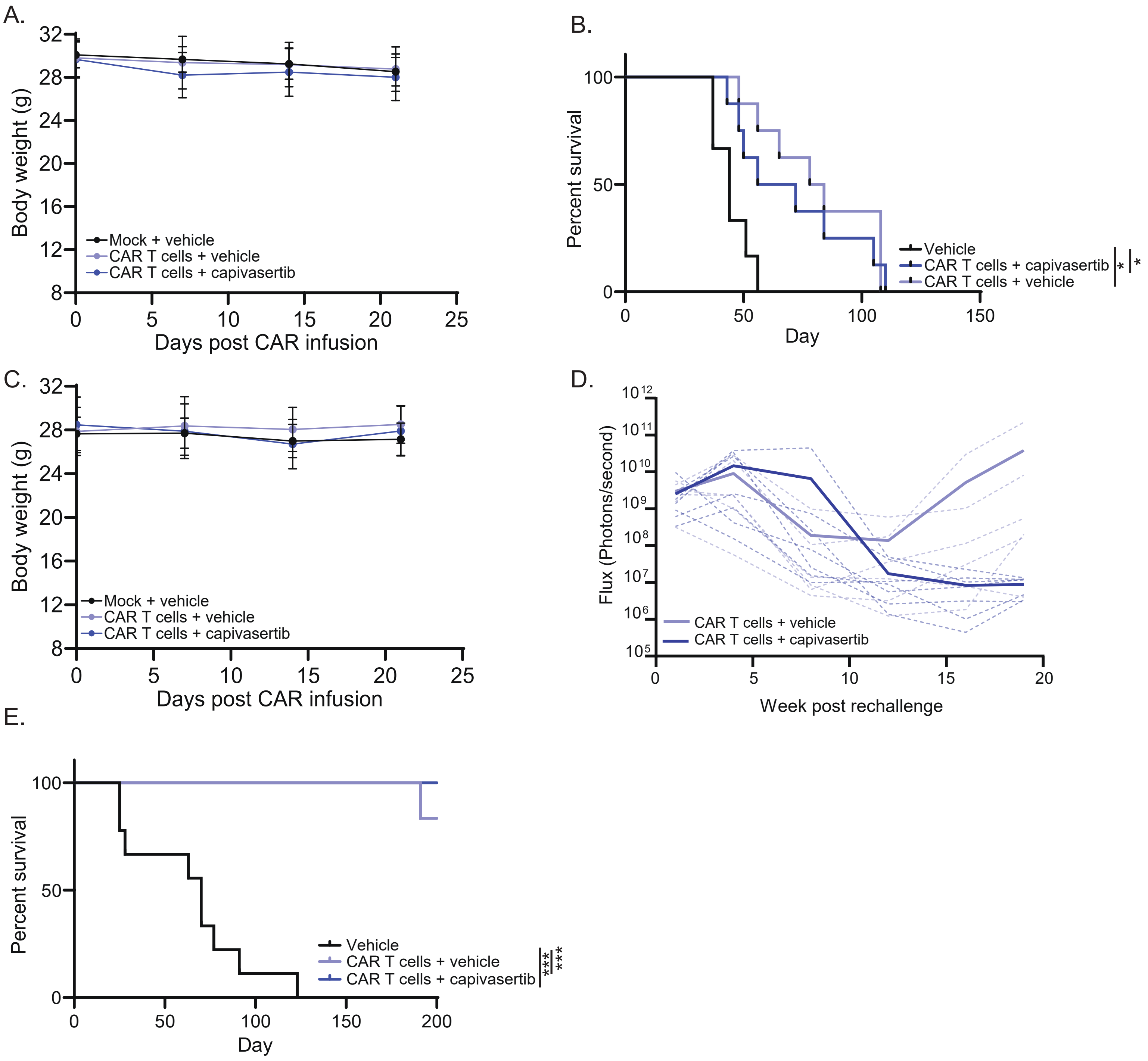

Figure S5. Combination of capivasertib and CAR T cells prevented relapse in Capiva-sertib susceptible model. Day 0 marked the initiation of treatment, with mice receiving a single dose of T cells and a 2-week regimen of either vehicle or capivasertib. (A) Mice bearing JeKo-1 cells were weighed once a week for 3 weeks. (B) Kaplan Meier survival curve of capivasertib resistant model (JeKo-1). (C) Mice bearing BJAB cells were weighed once a week for 3 weeks. (D) On day 67, post-tumor engraftment, mice were rechallenged with 2 x 10^6 capivasertib susceptible BJAB cells intratibially and analyzed for tumor growth by live mice imaging. (E) Kaplan Meier survival cruve. n = 8 (mock T cell groups) & 9 (CAR T cell groups) mice per experiment in the JeKo-1 model and n = 6 & 7 (the CAR T cells + capivasertib group) mice per experiment in the BJAB model, error bar: mean ± SD. * $p<0.05$, *** $p<0.001$.
